# New Insights into the Roles of Host Gene-Necrotrophic Effector Interactions in Governing Susceptibility of Durum Wheat to Tan Spot and Septoria nodorum Blotch

**DOI:** 10.1534/g3.116.036525

**Published:** 2016-10-24

**Authors:** Simerjot K. Virdi, Zhaohui Liu, Megan E. Overlander, Zengcui Zhang, Steven S. Xu, Timothy L. Friesen, Justin D. Faris

**Affiliations:** * Department of Plant Sciences, North Dakota State University, Fargo, North Dakota 58108; † Department of Plant Pathology, North Dakota State University, Fargo, North Dakota 58108; ‡ United States Department of Agriculture-Agricultural Research Service, Cereal Cops Research Unit, Northern Crop Science Laboratory, Fargo, North Dakota 58102

**Keywords:** durum wheat, tan spot, Septoria nodorum, necrotrophic pathogen, disease resistance

## Abstract

Tan spot and Septoria nodorum blotch (SNB) are important diseases of wheat caused by the necrotrophic fungi *Pyrenophora tritici-repentis* and *Parastagonospora nodorum*, respectively. The *P. tritici-repentis* necrotrophic effector (NE) Ptr ToxB causes tan spot when recognized by the *Tsc2* gene. The NE ToxA is produced by both pathogens and has been associated with the development of both tan spot and SNB when recognized by the wheat *Tsn1* gene. Most work to study these interactions has been conducted in common wheat, but little has been done in durum wheat. Here, quantitative trait loci (QTL) analysis of a segregating biparental population indicated that the *Tsc2*-Ptr ToxB interaction plays a prominent role in the development of tan spot in durum. However, analysis of two biparental populations indicated that the *Tsn1*-ToxA interaction was not associated with the development of tan spot, but was strongly associated with the development of SNB. *Pa. nodorum* expressed *ToxA* at high levels in infected *Tsn1* plants, whereas *ToxA* expression in *P. tritici-repentis* was barely detectable, suggesting that the differences in disease levels associated with the *Tsn1*-ToxA interaction were due to differences in pathogen expression of *ToxA*. These and previous results together indicate that: (1) the effects of *Tsn1*-ToxA on tan spot in common wheat can range from nonsignificant to highly significant depending on the host genetic background; (2) *Tsn1*-ToxA is not a significant factor for tan spot development in durum wheat; and (3) *Tsn1*-ToxA plays a major role in SNB development in both common and durum wheat. Durum and common wheat breeders alike should strive to remove both *Tsc2* and *Tsn1* from their materials to achieve disease resistance.

Durum wheat [*Triticum turgidum* ssp. *durum* (Desf.) Husnot.], also known as pasta or macaroni wheat, is an allotetraploid (2*n* = 4*x* = 28, AABB genomes) of worldwide economic importance because it is used to make pasta and other semolina-based products. Durum wheat production is affected by numerous diseases. Among some of the most severe are the foliar diseases tan spot and SNB caused by the necrotrophic fungal pathogens *P. tritici-repentis* and *Pa. nodorum*, respectively. Both pathogens are members of the Pleosporales order of fungi and are known to produce NEs (Oliver *et al.* 2012; Faris *et al.* 2013 for reviews). When a specific NE is recognized by the corresponding host gene, a host “defense response” ensues, which leads to programmed cell death allowing these necrotrophs to penetrate, feed, and sporulate. The lack of NE recognition by the host leads to resistance. Therefore, these host–pathogen interactions operate in an inverse gene-for-gene manner (Wolpert *et al.* 2002; Friesen and Faris 2010; Oliver *et al.* 2012; Faris *et al.* 2013), and the dominant alleles of the host NE recognition genes are considered susceptibility genes.

*P. tritici-repentis* is known to produce at least three NEs including Ptr ToxA, Ptr ToxB, and Ptr ToxC, and these NEs are recognized by the host genes *Tsn1*, *Tsc2*, and *Tsc1*, which reside on wheat chromosome arms 5BL, 2BS, and 1AS, respectively (see Singh *et al.* 2010; Faris *et al.* 2013 for reviews). Of the three NEs, the molecular nature of Ptr ToxA and Ptr ToxB have been determined (Ciuffetti *et al.* 2010 for review) and both are small secreted proteins that induce necrosis and chlorosis in wheat lines harboring *Tsn1* and *Tsc2*, respectively (Faris *et al.* 2013). Of the host genes, only *Tsn1* has been cloned, and it has features resembling classic disease resistance genes include protein kinase, nucleotide binding, and leucine-rich repeat domains (Faris *et al.* 2010).

The *Tsn1*-Ptr ToxA, *Tsc2*-Ptr ToxB, and *Tsc1*-Ptr ToxC interactions have all been shown to play significant roles in the development of tan spot in common (hexaploid) wheat (*T. aestivum* L. ssp. *aestivum*, 2*n* = 6*x* = 42, AABBDD genomes) (Faris *et al.* 2013; Kariyawasam *et al.* 2016). These experiments were conducted by infiltrating leaves of wheat lines from segregating populations with cultures containing crude, partially purified, or purified cultures of the NEs and inoculating the same wheat lines with conidia produced by the fungus to evaluate the development of tan spot. Many studies showed statistically significant relationships between sensitivity to the NEs and susceptibility to *P. tritici-repentis*. For example, early work with Ptr ToxA showed strong correlations between sensitivity to culture filtrates containing Ptr ToxA and susceptibility to Ptr ToxA-producing isolates (Tomas and Bockus 1987; Lamari and Bernier 1989). Similarly, studies using Ptr ToxB and Ptr ToxC showed that these NEs were also strongly associated with tan spot caused by isolates that produced them (Effertz *et al.* 2002; Friesen and Faris 2004; Abeysekara *et al.* 2010). Therefore, the NEs were considered virulence factors, and it was assumed that sensitivity to an NE would lead to disease susceptibility (Anderson *et al.* 1999). This led to the notion that lines could be screened with NE-containing cultures to more or less predict their reaction to tan spot. However, more recent studies, particularly involving the *Tsn1*-Ptr ToxA interaction, indicated that NE sensitivity did not always define tan spot susceptibility, and the involvement of the *Tsn1*-Ptr ToxA interaction in the development of disease was dependent on the genetic background of the host (Friesen *et al.* 2003; Faris and Friesen 2005; Chu *et al.* 2008a; Faris *et al.* 2012; Kariyawasam *et al.* 2016).

The vast majority of studies involving tan spot have been conducted in common wheat and relatively few have been conducted in durum wheat. P. K. Singh *et al.* (2008) showed that two linked recessive genes on chromosome arm 3BL conferred resistance to race 3 (produces Ptr ToxC) and race 5 (produces Ptr ToxB) isolates in tetraploid wheat, indicating that the *Tsc1*-Ptr ToxC and *Tsc2*-Ptr ToxB interactions were not involved. In another tetraploid wheat mapping study, Chu *et al.* (2010a) evaluated a durum doubled haploid population that segregated for Ptr ToxA sensitivity with race 1 (produces Ptr ToxA and Ptr ToxC) and race 2 (produces Ptr ToxA) isolates, and found that neither the *Tsn1*-Ptr ToxA nor the *Tsc1*-Ptr ToxC interaction was relevant in the development of disease. Therefore, although the three host gene-NE interactions in the wheat-*P. tritici-repentis* system have been shown to play variable roles in disease development in hexaploid wheat, none of them have been shown to be associated with the development of tan spot in durum wheat thus far.

To date, nine interactions involving specific wheat genes and cognate NEs have been reported in the wheat-*Pa. nodorum* system, and all have been shown to play significant roles in the development of SNB (Liu *et al.* 2004a,b, 2006; Friesen *et al.* 2006, 2007, 2008, 2009, 2012; Abeysekara *et al.* 2009, 2012; Chu *et al.* 2010b; Zhang *et al.* 2011; Gao *et al.* 2015; Shi *et al.* 2015). One of these interactions is the *Tsn1*-SnToxA (hereafter, both Ptr ToxA and SnToxA will be referred to as ToxA) interaction. Friesen *et al.* (2006) showed that the *ToxA* gene was horizontally transferred from *Pa. nodorum* to *P. tritici-repentis* sometime prior to 1940. This event likely played a role in tan spot becoming an economically significant disease, and henceforth *Tsn1* operated as a susceptibility gene for both tan spot and SNB. Liu *et al.* (2006) showed that the ToxA proteins derived from both *P. tritici-repentis* and *Pa. nodorum* functioned in the same way to elicit cell death when the proteins were infiltrated into wheat leaves. Numerous studies have shown that the *Tsn1*-ToxA interaction plays a major role in conferring susceptibility to *Pa. nodorum* in hexaploid wheat (Friesen *et al.* 2006, 2007, 2008, 2009, 2012; Chu *et al.* 2010b), and at least two studies have demonstrated the prominence of *Tsn1* in conferring susceptibility in tetraploid wheat (Faris and Friesen 2009; Friesen *et al.* 2012). Therefore, unlike for tan spot, *Tsn1* has been shown to be an important SNB susceptibility gene in both hexaploid and tetraploid wheat.

The primary objectives of this research were to determine the roles of the *Tsn1*-ToxA and *Tsc2*-Ptr ToxB interactions in a biparental population derived from two durum varieties. We also evaluated the role of the *Tsn1*-ToxA interaction in governing tan spot susceptibility in a second tetraploid wheat population, which was the same population used by Faris and Friesen (2009) to show that the interaction explained 95% of the variation in the development of SNB. Finally, we evaluated *ToxA* transcription in plants inoculated with *Pa. nodorum* or *P. tritici-repentis* to determine whether *ToxA* expression levels were correlated with the levels of disease caused by these two pathogens.

## Materials and Methods

### Plant materials

**Figure 1:**
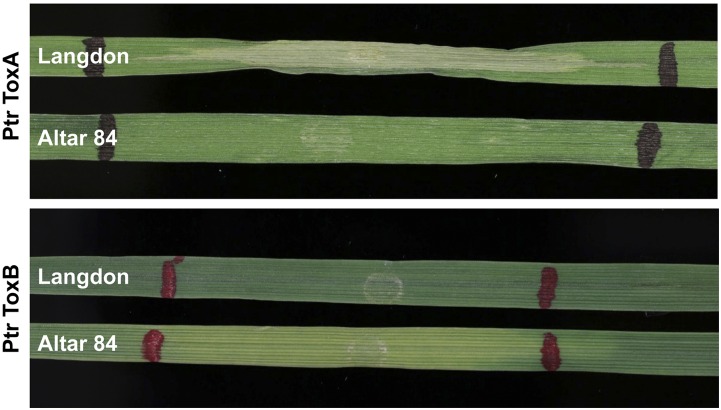
Leaves of Langdon and Altar 84 infiltrated with ToxA and Ptr ToxB. Langdon is sensitive to necrosis caused by ToxA and Altar 84 is insensitive, whereas Langdon is insensitive to the chlorosis caused by Ptr ToxB and Altar 84 is sensitive.

A segregating population of 127 recombinant inbred lines (RILs) was developed from a cross between the CYMMIT-bred durum variety “Altar 84” and the North Dakota durum variety “Langdon” (LDN). The RILs were developed by advancing the plants to the F_7_ generation by single seed descent (SSD). Preliminary experiments indicated that Altar 84 was sensitive to Ptr ToxB and insensitive to ToxA and Langdon was sensitive to ToxA and insensitive to Ptr ToxB, respectively (Figure 1). This population is hereafter referred to as the AL population.

The second population was derived from a cross between LDN and LDN-DIC 5B, which is a genetic stock where a pair of 5B chromosomes derived from the *T. turgidum* ssp. *dicoccoides* accession Israel A was substituted for the native 5B chromosomes in the LDN background (Joppa 1993). This population, hereafter referred to as the LD5B population, consisted of 85 recombinant inbred chromosome lines (RICLs) and was used by Faris and Friesen (2009) to evaluate the role of the *Tsn1*-ToxA interaction in conferring SNB susceptibility. Here, it was used to evaluate the role of the *Tsn1*-ToxA interaction in conferring susceptibility to tan spot caused by the race 2 (ToxA producing) isolate 86–124.

All plants were grown in cones containing SB100 (Sun Gro Sunshine; Sun Gro Horticulture, Vancouver, BC) soil mix with 10–20 granules of Osmocote fertilizer (Scotts Company LLC, Marysville, OH) added to each cone. For disease evaluations and NE infiltrations, all plants were grown in the greenhouse at an average temperature of 21° with a 16 hr photoperiod.

### NE production and infiltration assays

ToxA and Ptr ToxB were expressed in the yeast strain *Pichia pastoris* X33 and cultured as described for the NE SnTox3 in Liu *et al.* (2009). Harvested cultures were used to directly infiltrate the second leaf of wheat plants at the two-leaf stage. Infiltrations were conducted using a 1 ml needleless syringe on the secondary leaf until 2–3 cm of leaf was infiltrated. The boundaries of the infiltrated region were marked using a nontoxic permanent marker. The infiltrated plants were then placed in a growth chamber at 21° with a 16 hr photoperiod. Plants were evaluated 4 d after infiltration and scored as sensitive or insensitive based on the presence or absence of chlorosis for Ptr ToxB or necrosis for ToxA. The entire AL population and parents were infiltrated twice with ToxA and three times with Ptr ToxB. The infiltration scores, *i.e.*, insensitive *vs.* sensitive, were converted to genotypic scores for placement of the *Tsn1* and *Tsc2* loci on the genetic linkage maps relative to the molecular markers. For the LD5B population, the reaction of each line to ToxA and the placement of the *Tsn1* locus on the genetic linkage map were previously determined (Faris and Friesen 2009).

### Disease evaluations

For *P. tritici-repentis* disease evaluation, the AL population and parents were screened with race 2 isolates 86–124 and L13-35, which produce ToxA, and the race 5 isolate DW5 which produces Ptr ToxB. The LD5B population was screened with 86–124. Isolates were grown on V8-potato dextrose agar (Difco PDA; Becton, Dickinson and Company, Sparks, MD) plates for 5–7 d in the dark, and inoculum was prepared as described in Lamari and Bernier (1989) and Ali *et al.* (2010). Parents and the RIL population were planted in a completely randomized design consisting of three replicates separated by time for conidial inoculations. Each replicate consisted of a single cone per line with three plants per cone placed in racks of 98 cones. The tan spot-susceptible wheat variety “Jerry” was planted in the borders of each rack to reduce edge effects. Plants were inoculated until runoff at the two- to three-leaf stage with 3000 spores per ml and two drops of Tween 20 (polyoxyethylene sorbitan monolaurate; J.T. Baker Chemical Co., Phillipsburg, NJ) per 100 ml of inoculum. Inoculated plants were placed in a mist chamber with 100% relative humidity at 21° for 24 hr, and then moved to a growth chamber for 6 d of incubation at 21° under a 12 hr photoperiod. Inoculated plants were rated using a 1–5 lesion type scale (Lamari and Bernier 1989) at 7 d postinoculation, where one is resistant and five is susceptible.

For *Pa. nodorum* disease evaluations, the AL population was screened with *Pa. nodorum* isolate Sn2000, which is known to produce ToxA (Friesen *et al.* 2006). Inoculum production and inoculation procedures were done as described in Liu *et al.* (2004a). After inoculation, plants were placed in a mist chamber with 100% relative humidity at 21° for 24 hr, and then moved to a growth chamber at 21° with a 12 hr photoperiod. Disease evaluation was carried out at 7 d after inoculation by scoring lesions on the second leaf using the 0–5 scale described by Liu *et al.* (2004).

### Marker genotyping

The AL population and parents were genotyped using the iSelect array containing 9000 wheat single nucleotide polymorphism (SNP) markers (Cavanagh *et al.* 2013) as described in Faris *et al.* (2014). Simple sequence repeat (SSR) markers were also used to genotype the AL population and were selected from the following libraries: MAG (Xue *et al.* 2008), WMS (gwm) (Röder *et al.* 1998), WMC (Somers *et al.* 2004), HBG (Torada *et al.* 2006), CFD (Sourdille *et al.* 2004), and BARC (Song *et al.* 2005). SSR primer sets were used to amplify the parental DNA using polymerase chain reaction (PCR) conditions as outlined in Lu and Faris (2006). PCR amplifications were performed in 10 µl reactions consisting of 100 ng of DNA template, 1.5 mM MgCl_2_, 0.125 mM dNTPs, 4 pmol of primers, and 1 unit of *Taq* DNA polymerase. The PCR conditions were 94° for 4 min, followed by 35 cycles of 94° for 30 sec, the appropriate annealing temperature for 30 sec, and 72° for 1 min. The annealing temperature of each primer was obtained from the Graingenes website (http://wheat.pw.usda.gov/GG2/index.shtm). Fragments were electrophoresed on 6% polyacrylamide gels which were made using 46 ml of H_2_O, 6 ml 10 × TBE (Tris-borate-EDTA), 9 ml of 40% acrylamide/bis-acrylamide, 40 µl tetramethylethylenediamine (TEMED), and 350 µl of 10% ammonium persulfate. Gels were stained with GelRed (Biotium, Inc.) for 10 min and then visualized with a Typhoon 9410 variable mode imager (GE Healthcare, Waukesha, WI). Markers that revealed polymorphisms between the parents were then used to genotype the AL population.

In addition to the SSR and SNP markers, a cleaved amplified polymorphic sequence (CAPS) marker (*Xfcp667*) based on the genomic sequence of the *Snn1* gene (Shi *et al.* 2016) was used to map the *Snn1* locus. A fragment of the *Snn1* gene was PCR-amplified as described for the SSR markers above using an annealing temperature of 65° and the primers FCP667F: 5ʹ-TGCGTCGATAGGAGTG-3ʹ and FCP667R: 5ʹ-ATGGCGTAGGAGCACGGGTA-3ʹ. The 898 bp amplicon was then digested with the restriction enzyme *Hpy*CH4IV, which cleaves the Altar 84 fragment, but not the LDN fragment, thus revealing a codominant polymorphism. The digested amplicons were electrophoresed on 2% agarose gels, stained with ethidium bromide, and photographed.

### Linkage, QTL, and statistical analysis

Linkage analysis was conducted using the computer program MapDisto 1.8.1 (Lorieux 2012) to generate linkage maps. First, marker grouping was done by using the command “find groups” with a logarithm of odds (LOD) > 3.0 and an Rmax value = 30.0. The “order sequence,” “check inversions,” “ripple order,” and “drop locus” commands were then used to determine the best order for each group. The Kosambi mapping function (Kosambi 1944) was used to calculate linkage distances.

In the AL population, multiple interval mapping (MIM) was used to determine the effects of the *Tsn1* and *Tsc2* loci in causing disease and to identify additional QTL associated with disease using the computer program QGene v.4.3 (Joehanes and Nelson 2008). An LOD of 3.6 was declared as the threshold for QTL significance based on permutation tests of 1000 iterations for each trait. The homogeneity of variances among the three replicates was determined by Bartlett’s χ^2^ test using SAS program version 9.4 (SAS Institute Inc., Cary, NC). Mean separation of the genotypic means were determined by Fisher’s protected LSD at an α level of 0.01. For the LD5B population, the critical LOD threshold of 1.8 declared in Faris and Friesen (2009) was used for determining significant associations for tan spot caused by 86–124. Because the LD5B population segregates for only one linkage group (5B), QTL analysis was conducted using the simple interval mapping (SIM) function in QGene v.4.3 (Joehanes and Nelson 2008).

### Gene expression analysis

Plants of LDN and LDN-DIC 5B were grown and inoculated with water, *P. tritici-repentis* isolate 86–124, or *Pa. nodorum* isolate Sn2000 as described above. The youngest fully expanded leaf at the time of inoculation was harvested for RNA extraction. Samples for each treatment of both genotypes were collected at 0, 6, 24, 48, 72, and 96 hr after inoculation and immediately frozen in liquid nitrogen. Total RNA was extracted using the RNeasy Plant Mini Kit (QIAGEN, Hilden, Germany), and first-strand cDNA was synthesized from 1 µg of total RNA using Taqman Reverse Transcription Reagents including an oligo d(T)_16_ primer (Applied Biosystems, Foster City, CA). Relative quantitative (RQ)-PCR was performed to evaluate *ToxA* gene expression using primers ToxA.RT.F3 (5ʹ-AACGCCAATACAGTGCGAGT-3ʹ) and ToxA.cod.1R (5ʹ-GCTGCATTCTCCAATTTTCACG-3ʹ) in all treatments and sampled time points. Expression of the *ToxA* gene was compared to the expression of the endogenous wheat ubiquitin gene as described in Faris *et al.* (2010) using primers Ta.Ubiquitin.F: 5ʹ-GCACCTTGGCGGACTACAACATTC-3ʹ and Ta.Ubiquitin.R: 5ʹ-GACACCGAAGACGAGACTTGTGAACC-3ʹ. All RQ-PCR experiments were conducted using a 7500 Real-Time PCR System (Applied Biosystems). Each experiment was conducted using three biological replicates each consisting of a single inoculated leaf, and at least three technical replicates per biological replicate were performed. The 10 μl PCR reactions contained 1 × SYBR PCR MasterMix (Applied Biosystems), 0.25 μM each primer, and 5 μl of 10-fold diluted cDNA. The thermocycler procedure was as follows: 10 min of preincubation at 95°, followed by 40 cycles for 15 sec at 95° and for 1 min at 60°. Efficiencies of the different primer combinations were evaluated using serial dilutions of cDNA (1:5, 1:10, 1:20, and 1:40) and only primers with efficiencies higher than 95% were used for the RQ-PCR. The expression level of the Sn2000-inoculated 48 hr sample was set at 1 as a calibration point. Threshold cycles of the *ToxA* gene and the endogenous ubiquitin gene were used to calculate the relative expression levels using the 2^−ΔΔCT^ method.

### Data availability

Parents and mapping populations are available upon request. Mapping data for the LD5B and the AL populations is also available upon request. The authors state that all data necessary for confirming the conclusions presented in the article are represented fully within the article.

## Results

### Marker analysis and linkage map construction

The parental lines of the AL population were screened with 250 SSR primer pairs. Of these, 119 (47.6%) revealed polymorphisms between the parents and were used to genotype the AL population. The 9K SNP array yielded a total of 833 polymorphic SNP markers. One CAPS marker (*Xfcp667*) that was developed based on the *Snn1* gene sequence on chromosome arm 1BS (Shi *et al.* 2016) was added to the marker set along with the two phenotypic markers *Tsn1* and *Tsc2* (see below). Therefore, the initial marker dataset consisted of a total of 955 markers. After initial linkage analysis, a total of 111 markers, including 19 SSRs and 92 SNPs, were eliminated from the dataset because they were unlinked, leaving a total of 844 markers in the dataset consisting of 100 SSRs, 741 SNPs, one CAPS, and two phenotypic (*Tsn1* and *Tsc2*) markers.

These markers were assembled into 14 linkage groups that corresponded to the 14 durum wheat chromosomes (Supplemental Material, Table S1) and spanned a total genetic distance of 2207.15 cM with an average marker density of one marker per 2.6 cM (Table 1). The A-genome chromosomes had 414 markers and spanned 1128.22 cM with an average density of one marker per 2.7 cM, whereas the B-genome chromosomes had a total of 430 markers spanning 1078.93 cM for an average marker density of one marker per 2.5 cM (Table 1). Chromosome 7A was the longest linkage group (262.41 cM) and chromosome 4B was the shortest (107.95 cM). The number of markers per chromosome ranged from 17 (4B) to 104 (5B) (Table 1 and Table S1). Chromosome 6B had the highest marker density at one marker per 1.2 cM, whereas chromosome 4B had the lowest with one marker every 6.3 cM (Table 1). Of the 844 markers, 208 (24.6%), had segregation ratios that deviated significantly (*P* < 0.05) from the expected 1:1 ratio. These distorted markers were located on 10 chromosomes (1A, 2A, 2B, 3B, 4B, 5A, 5B, 6A, 6B, and 7B).

**Table 1:** Summary of markers mapped in each chromosome/genome in the Altar 84 × Langdon population

Chromosome	Markers			Phenotype	Total	Length (cM)	Marker Density (cM/Marker)	Markers with Distorted Ratios
	SSR	CAPS	SNP					
1A	4	−	65	−	69	144.49	2.1	0
1B	20	1	53	−	74	159.84	2.2	12
2A	4	−	57	−	61	178.68	2.9	2
2B	9	−	36	1	46	196.73	4.2	22
3A	6	−	47	−	53	159.97	3.0	0
3B	3	−	31	−	34	167.19	4.9	9
4A	5	−	50	−	55	128.57	2.3	0
4B	2	−	15	−	17	107.95	6.3	8
5A	6	−	52	−	58	130.76	2.3	26
5B	23	−	80	1	104	211.72	2.0	71
6A	3	−	43	−	46	123.34	2.7	2
6B	7	−	88	−	95	111.10	1.2	52
7A	6	−	66	−	72	262.41	3.6	0
7B	2	−	58	−	60	124.40	2.1	4
A genome	34	−	380	−	414	1128.22	2.7	30
B genome	66	1	361	2	430	1078.93	2.5	178
Total	100	1	741	2	844	2207.15	2.6	208

SSR, simple sequence repeat; CAPS, cleaved amplified polymorphic sequence; SNP, single nucleotide polymorphism.

### Genetic analysis of ToxA and Ptr ToxB sensitivity in the AL population

**Figure 2:**
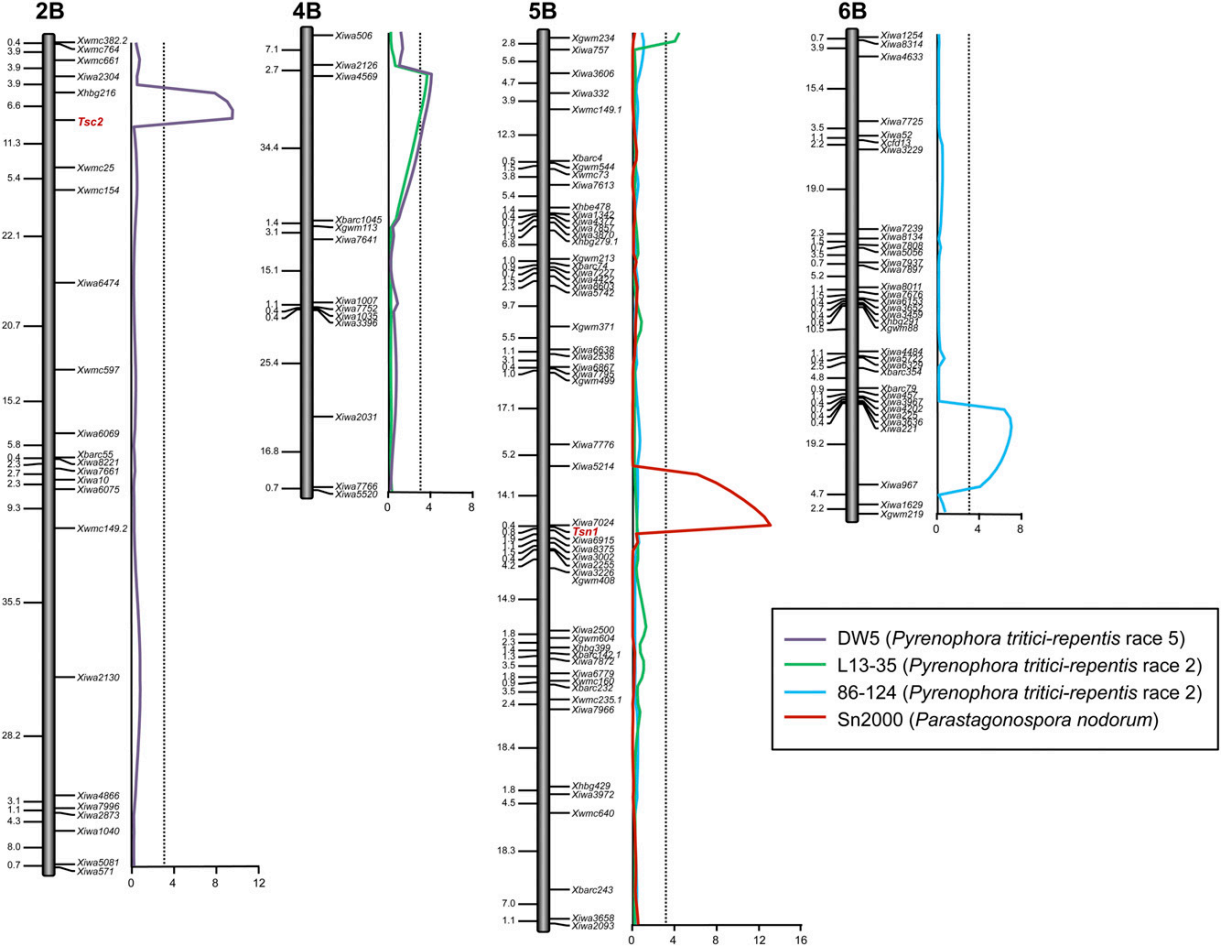
Composite interval regression maps of QTL associated with the *P. tritici-repentis* race 5 isolate DW5, *P. tritici-repentis* race 2 isolates L13-25 and 86-124, and the *Pa. nodorum* isolate Sn2000 in the Altar 84 × Langdon recombinant inbred population. Markers are indicated to the right side of the genetic maps and cM distances are shown along the left side. The critical LOD threshold is indicated by the dotted lines and the LOD scale is indicated at the bottom along the *x*-axis. LOD, logarithm of odds; QTL, quantitative trait loci.

Altar 84 was insensitive and Langdon was sensitive to ToxA (Figure 1). The AL population segregated in a ratio of 59 insensitive:68 sensitive for reaction to ToxA and fitted the expected 1:1 ratio for a single host gene conferring sensitivity to ToxA (χ^2^_df = 1_ = 0.65, *P* = 0.42). Conversion of the ToxA reaction scores to genotypic scores allowed us to map the *Tsn1* locus, which was located on the long arm of chromosome 5B as expected and flanked by SNP markers *Xiwa7024* and *Xiwa6915* at distances of 0.4 and 0.8 cM, respectively (Figure 2 and Table S1).

For reaction to Ptr ToxB, Altar 84 was sensitive and Langdon was insensitive (Figure 1). The AL population segregated in a ratio of 72 insensitive:55 sensitive for reaction to Ptr ToxB and fitted the expected 1:1 ratio for a single host gene conferring sensitivity to Ptr ToxB (χ^2^_df = 1_ = 3.28, *P* = 0.07). The reactions to Ptr ToxB were converted to genotypic scores and analyzed along with the molecular marker data. Linkage analysis showed that the *Tsc2* locus mapped to the short arm of chromosome 2B flanked by SSR markers *Xhbg216* and *Xwmc25* at distances of 6.6 and 11.3 cM, respectively (Figure 2 and Table S1).

### AL population reaction to P. tritici-repentis

Altar 84, LDN, and the AL population were screened with the ToxA-producing race 2 isolates 86–124 and L13-35, and the race 5 Ptr ToxB-producing isolate DW5. Bartlett’s Chi-squared test for homogeneity indicated that the variances among replicates for each isolate were not significantly different (86–124: χ^2^_df = 2_ = 2.08, *P* = 0.35; L13-35: χ^2^_df = 2_ = 1.39, *P* = 0.50; DW5: χ^2^_df = 2_ = 6.01, *P* = 0.05); therefore, the means from the three replicates for each isolate were used for further analysis.

**Figure 3:**
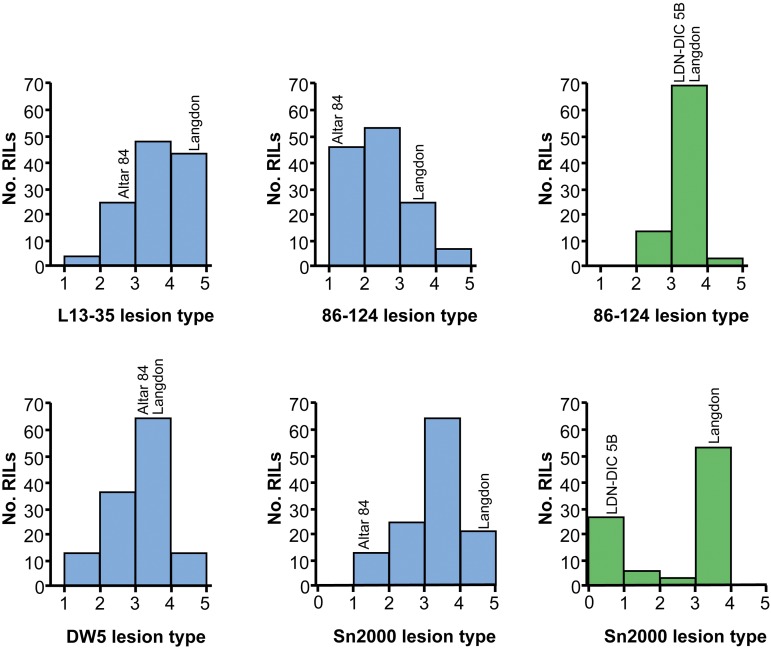
Histograms of average lesion type reactions of the Altar 84 × Langdon recombinant inbred population to *P. tritici-repentis* race 2 isolates L13-35 and 86-124, race 5 isolate DW5, and the *Pa. nodorum* isolate Sn2000 (blue bars), and of average lesion type reactions of the Langdon × LDN-DIC 5B recombinant inbred chromosome line population to *P. tritici-repentis* race 2 isolate 86–124 and the *Pa. nodorum* isolate Sn2000 (Faris and Friesen 2009) (green bars). RILs, recombinant inbred lines.

**Figure 4:**
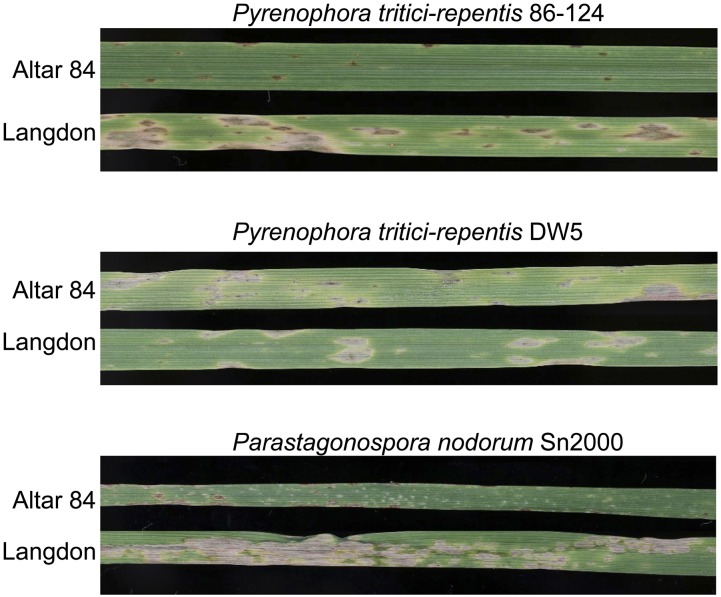
Leaves of Altar 84 and Langdon inoculated with *P. tritici-repentis* isolates 86–124 (race 2) and DW5 (race 5), and with *Pa. nodorum* isolate Sn2000.

Altar 84 and LDN were considered resistant and moderately susceptible to 86–124 with average disease reaction types of 1.17 and 3.50, respectively (Figure 3, Figure 4, and Table 2). The average disease reaction type for the AL population was 2.31 and reaction types ranged from 1.00 to 4.16 (Figure 3 and Table 2). The mean reaction types of ToxA-insensitive and -sensitive AL lines were 2.21 and 2.31, which were not significantly different at the 0.01 level of probability (Table 2), indicating that the *Tsn1*-ToxA interaction was not significant in the development of tan spot caused by 86–124.

**Table 2:** Average reaction types of Altar 84, Langdon, and the Altar 84 × Langdon population of recombinant inbred lines to tan spot and Septoria nodorum blotch

Isolate*^a^*	Altar 84	Langdon	AL Population Range	AL Population Average	ToxA Sensitive Lines	ToxA Insensitive Lines	Ptr ToxB Sensitive Lines	Ptr ToxB Insensitive Lines	Difference Between Sensitive and Insensitive Lines
86-124 (race 2)	1.17	3.50	1.00–4.16	2.31	2.39	2.21	−	−	0.18
L13-35 (race 2)	2.75	4.67	1.33–4.83	3.52	3.64	3.36	−	−	0.28
DW5 (race 5)	3.16	3.50	1.25–4.50	3.02	−	−	2.29	1.33	0.96*
Sn2000	1.33	4.83	1.16–4.80	3.18	3.00	2.00	−	−	1.00*

AL, Altar 84 × Langdon. *P < 0.01.

a Reaction types caused by the *P. tritici-repentis* isolates (86–124, L13-35, and DW5) were scored using the 1–5 scale for tan spot described by Lamari and Bernier (1989), and reaction types caused by the *Pa. nodorum* isolate (Sn2000) were scored using the 0–5 scale for Septoria nodorum blotch described by Liu *et al.* (2004).

For the other *P. tritici-repentis* race 2 isolate, L13-35, Altar 84, and LDN were moderately susceptible and susceptible with average reaction types of 2.75 and 4.67, respectively (Figure 3 and Table 2). The AL population reaction types averaged 3.52 and ranged from 1.33 to 4.83 indicating some transgressive segregation for resistance (Figure 3 and Table 2). Average L13-35 reaction types for ToxA-sensitive and -insensitive AL lines were 3.64 and 3.36, respectively, which were not significantly different at the 0.01 level of probability (Table 2), again suggesting that the *Tsn1*-ToxA interaction did not play a significant role in the development of tan spot in the AL population.

For reaction to the race 5 Ptr ToxB-producing isolate DW5, Altar 84 and LDN were both moderately susceptible with average disease reactions of 3.16 and 3.50, respectively (Figure 3, Figure 4, and Table 2). The average disease reaction type for the AL population was 3.02, and reaction types ranged from 1.25 to 4.50 (Figure 3 and Table 2), indicating strong transgressive segregation and suggesting that more than one gene was involved in conditioning resistance. The mean reaction types of Ptr ToxB-sensitive and -insensitive AL lines to DW5 were 2.29 and 1.33, respectively, which were significantly different at the 0.01 level of probability (Table 2). This result indicates that the *Tsc2*-Ptr ToxB interaction played a significant role in the development of tan spot caused by DW5.

### AL population reaction to Pa. nodorum

As with the *P. tritici-repentis* isolates, Bartlett’s Chi-squared test for homogeneity of variances among *Pa. nodorum* isolate Sn2000 replicates was not significant (χ^2^_df = 2_ = 0.78, *P* = 0.70), and therefore the means of the three reps were used for further analysis. Altar 84 was resistant to Sn2000 and LDN was highly susceptible with average disease reaction types of 1.33 and 4.83, respectively (Figure 3 and Table 2). The average disease reaction type for the AL population was 3.18, and reaction types ranged from 1.16 to 4.80 (Figure 3 and Table 2). The mean reaction types of ToxA-insensitive and -sensitive AL lines were 2.00 and 3.00, respectively (Table 2). These means were significantly different at the 0.01 level of probability (Table 2), indicating that the *Tsn1*-ToxA interaction played a significant role in the development of SNB caused by Sn2000.

### QTL analysis of the AL population for reaction to P. tritici-repentis and Pa. nodorum

For the race 2 isolate 86–124, only one QTL was significantly associated with resistance (Figure 2 and Table 3). This QTL was on the long arm of 6B between markers *Xiwa5148* and *Xiwa967*, which were at positions 84.4 and 103.4 cM, respectively. This QTL, designated *QTs.fcu-6B*, had an LOD of 6.9 and explained 22% of the phenotypic variation (Table 3). The resistance effects of *QTs.fcu-6B* were contributed by Altar 84 (Table 3).

**Table 3:** Composite interval mapping analysis of QTL associated with resistance to tan spot caused by *P. tritici-repentis* races 2 and 5 and resistance to SNB caused by Sn2000 in the Altar 84 × Langdon (AL) population

QTL	Marker Interval	Marker Position	86-124 (*P. tritici-repentis* Race 2)			L13-35 (*P. tritici-repentis* Race 2)			DW5 (*P. tritici-repentis* Race 5)			Sn2000 (*Pa. nodorum*)		
			LOD	*R^2^*	Add.	LOD	*R^2^*	Add.	LOD	*R^2^*	Add.	LOD	*R^2^*	Add.
*QTs.fcu-2B*	*Tsc2*	18.7	–	–	–	–	–	–	12.00	0.26	0.41	–	–	–
*QTs.fcu-4B*	*Xiwa2126-Xgwm113*	7.1-38.9	–	–	–	4.00	0.11	−0.37	8.60	0.12	−0.56	–	–	–
*QTs.fcu-5B*	*Xgwm234*	0.0	–	–	–	4.20	0.12	−0.31	–	–	–	–	–	–
*QTs.fcu-6B*	*Xiwa5148-Xiwa967*	84.4–103.4	6.90	0.22	−0.51	–	–	–	–	–	–	–	–	–
*QSnb.fcu-5B*	*Tsn1*	110.2	–	–	–	–	–	–	–	–	–	13.00	0.38	−0.59

A negative value for “Add.” indicates resistance effects derived from Altar 84. A dash indicates that the marker was not significantly associated with resistance. QTL, quantitative trait loci; LOD, logarithm of odds; Add., the additive effects of the QTL.

Two significant QTL were associated with the other *P. tritici-repentis* race 2 isolate, L13-35 (Figure 2). QTL on the short arms of chromosomes 4B and 5B, designated *QTs.fcu-4B* and *QTs.fcu-5B*, had LOD values of 4.0 and 4.2, and explained 11 and 12% of the phenotypic variation, respectively (Table 3). The resistance effects at both of these QTL were contributed to by Altar 84. The *Tsn1* locus on 5BL was not significantly associated with resistance to either of the two race 2 isolates 86–124 and L13-35 (Figure 2), which both produce ToxA. In single factor regression, *Tsn1* had LOD values of only 0.35 and 0.40 for 86–124 and L13-35, respectively.

**Figure 5:**
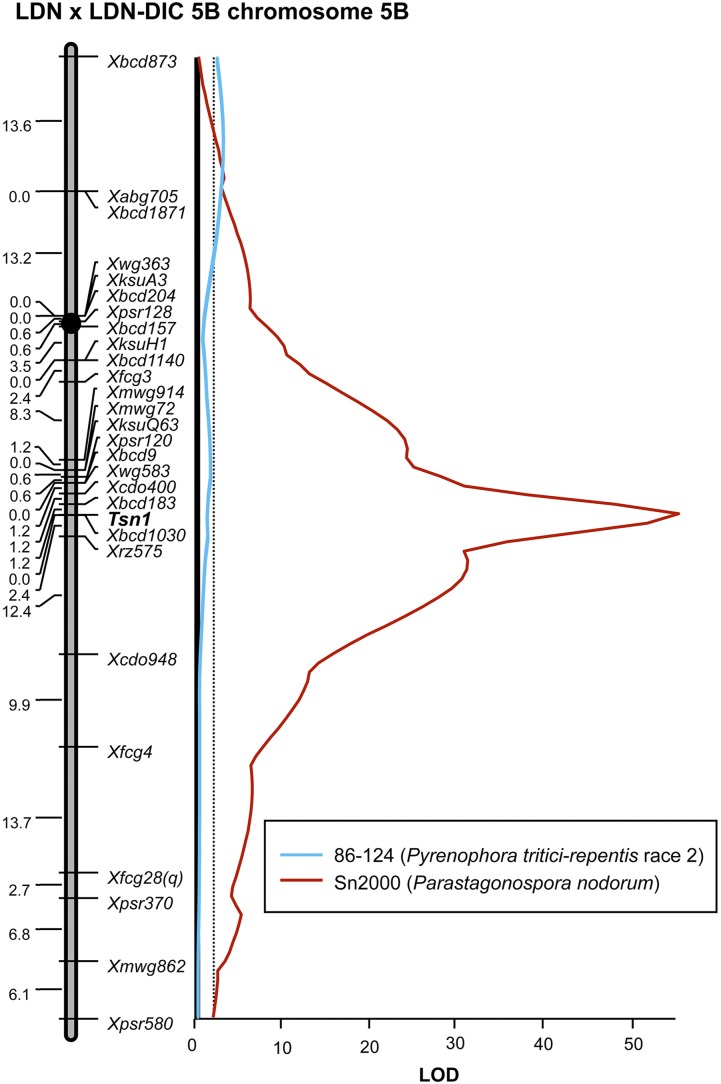
Interval regression maps of QTL associated with reaction to the *P. tritici-repentis* race 2 isolate 86–124 and the *Pa. nodorum* isolate Sn2000 in the Langdon × LDN-DIC 5B recombinant inbred chromosome line population. Markers are indicated to the right side of the genetic maps and cM distances are shown along left side. The critical LOD threshold is indicated by the dotted lines and the LOD scale is indicated at the bottom along the *x*-axis. The linkage map and Sn2000 regression analysis were first reported in Faris and Friesen (2009). LOD, logarithm of odds; QTL, quantitative trait loci.

For the race 5 isolate DW5, two significant QTL were identified. The QTL were located on chromosome arms 2BS and 4BL and designated *QTs.fcu-2B* and *QTs.fcu-4B*, respectively (Figure 5 and Table 3). The peak of *QTs.fcu-2B* was defined by the *Tsc2* locus on chromosome 2BS. It had an LOD of 12.0 and explained 26% of the disease variation (Figure 2 and Table 3). Because this QTL represented the *Tsc2*-Ptr ToxB interaction for which Altar 84 contributed the Ptr ToxB-sensitive allele, resistance to this QTL was contributed by LDN (Table 3).

The *QTs.fcu-4B* QTL associated with reaction to tan spot caused by DW5 was that same QTL found to be associated with the *P. tritici-repentis* race 2 isolate L13-35 (Figure 2 and Table 3). In association with disease caused by DW5, *QTs.fcu-4B* had an LOD of 8.6 and explained 12% of the variation with resistance effects contributed by Altar 84 (Table 3). The QTL peaked between markers *Xiwa2126* and *Xgwm113*, which were located at positions 7.1 and 38.9 cM, respectively (Figure 2 and Table 3).

A single QTL, designated *QSnb.fcu-5B*, associated with SNB caused by the ToxA-producing *Pa. nodorum* isolate Sn2000 was identified on the long arm of 5B (Figure 2). The *Tsn1* locus, with an LOD of 13.0, defined the peak of *QSnb.fcu-5B* and explained 38% of the phenotypic variation (Figure 2 and Table 3). Because this QTL is due to the effects of the *Tsn1*-ToxA interaction, it indicates that this interaction played a significant role in the development of SNB caused by isolate Sn2000.

### LD5B population reaction to P. tritici-repentis and Pa. nodorum

Bartlett’s Chi-squared test for homogeneity among the three replicates of the LD5B population inoculated with *P. tritici-repentis* isolate 86–124 indicated they were homogeneous (χ^2^_df = 2_ = 0.12, *P* = 0.94), and therefore the means of the three replicates were calculated and used for further analysis. LDN and LDN-DIC 5B had mean reaction types of 3.60 and 3.00, respectively, to 86–124 (Table 4). The LD5B population reaction types ranged from 2.25 to 4.00 with an average of 3.16 (Figure 3 and Table 4). ToxA-sensitive and -insensitive lines had average reaction types of 3.16 and 3.04 in response to 86–124, which were not significantly different (Table 4), indicating that the *Tsn1*-ToxA interaction played no role in the development of disease caused by *P. tritici-repentis* race 2 isolate 86–124.

**Table 4:** Average reaction types of Langdon, LDN-DIC 5B, and the Langdon × LDN-DIC 5B (LD5B) population of recombinant inbred chromosome lines to tan spot and Septoria nodorum blotch

Isolate	Langdon	LDN-DIC 5B	LD5B Population	LD5B Population Average	ToxA Sensitive Lines	ToxA Insensitive Lines	Difference Between Sensitive and Insensitive Lines
86-124	3.60	3.00	2.25–4.00	3.16	3.16	3.04	0.12
Sn2000*^a^*	3.50	0.83	0.33–3.83	2.40	3.24	0.78	2.46*

* *P* < 0.01.

a The data and analysis of Sn2000 in the LD5B population was taken from Faris and Friesen (2009).

As mentioned above, we previously reported the analysis of the LD5B population for reaction to *Pa. nodorum* isolate Sn2000 (Faris and Friesen 2009). In that study, we showed that LDN and LDN-DIC 5B had average reaction types of 3.50 and 0.83, respectively. The LD5B population had an overall average reaction type of 2.40 and ranged from 0.33 to 3.83 (Figure 3 and Table 4) (Faris and Friesen 2009). Also, ToxA-sensitive lines were highly susceptible to Sn2000 with an average reaction type of 3.24, whereas ToxA-insensitive lines were highly resistant with an average reaction type of 0.78, indicating that the *Tsn1*-ToxA interaction was a major factor in determining susceptibility to the *Pa. nodorum* isolate Sn2000 (Faris and Friesen 2009).

### QTL analysis of the LD5B population

A QTL with an LOD value of 3.8, explaining 19% of the phenotypic variation, was found to be associated with tan spot caused by the *P. tritici-repentis* race 2 isolate 86–124 (Figure 5 and Table 5). This QTL was located in the distal region of chromosome arm 5BS and may be the same as *QTs.fcu-5B*, which was associated with the *P. tritici-repentis* race 2 isolate L13-35 in the AL population. As with *QTs.fcu-5B* in the AL population, the susceptible allele at this locus was contributed to by LDN (Table 5). The *Tsn1* locus was not significantly associated with tan spot caused by 86–124 in this population. On the contrary, we previously showed that the *Tsn1* locus was a major factor in conferring susceptibility to SNB caused by the *Pa. nodorum* isolate Sn2000 (Faris and Friesen 2009). In that research, the *Tsn1* locus had an LOD of 54.0 and explained 95% of the phenotypic variation (Figure 5 and Table 5) (Faris and Friesen 2009).

**Table 5:** Simple interval mapping analysis of QTL associated with resistance to tan spot caused by *P. tritici-repentis* race 2 isolate 86–124 and to SNB caused by the *Pa. nodorum* isolate Sn2000 in the Langdon × LDN-DIC 5B (LD5B) population

QTL	Marker Interval	Marker Position	86-124 (*P. tritici-repentis* Race 2)			Sn2000 (*Pa. nodorum*)		
			LOD	*R^2^*	Add.	LOD	*R^2^*	Add.
*QTs.fcu-5B*	*Xbcd873-Xabg705*	0–13.6	3.80	0.19	−0.18	–	–	–
*QSnb.fcu-5B*	*Tsn1*	48.2	–	–	–	54.0	0.95	−1.30

A dash indicates that the marker was not significantly associated with resistance. A negative value indicates resistance effects derived from LDN-DIC 5B. QTL, quantitative trait loci; LOD, logarithm of odds; Add., the additive effects of the QTL.

### Expression analysis of ToxA in P. tritici-repentis- and Pa. nodorum-inoculated plants

**Figure 6:**
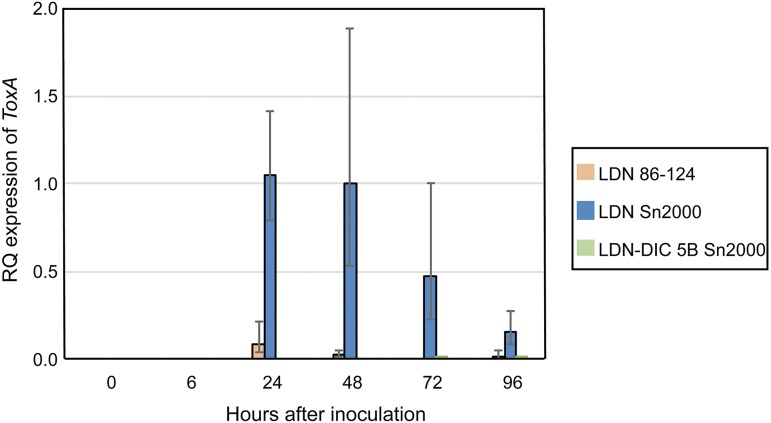
Relative quantitative transcriptional expression of the *ToxA* gene in the susceptible line Langdon inoculated with water, the *P. tritici-repentis* race 2 isolate 86–124, and the *Pa. nodorum* isolate Sn2000, and in the resistant line LDN-DIC 5B inoculated with the *Pa. nodorum* isolate Sn2000. The expression of the *ToxA* gene in each treatment was normalized to the expression of the wheat ubiquitin gene. For LDN-DIC 5B inoculated with Sn2000, *ToxA* transcripts were detected only at 72 and 96 hr postinoculation, and at levels that were barely visible on the graph compared to *ToxA* expression in Langdon inoculated with Sn2000. Expression of *ToxA* in the water-inoculated Langdon and LDN-DIC 5B plants and in LDN-DIC 5B plants inoculated with 86–124 was not detected, and therefore no bars representing these treatments are included on the graph. Expression of ToxA in the Langdon plants inoculated with Sn2000 was significantly greater (*P* < 0.01) than in 86–124 and water-inoculated Langdon plants for the 24, 48, 72, and 96 hr time points.

The susceptible/ToxA-sensitive line LDN and the resistant/ToxA-insensitive line LDN-DIC 5B were inoculated with water, the *P. tritici-repentis* race 2 isolate 86–124, and the *Pa. nodorum* isolate Sn2000, and samples were collected for RNA extraction at 0, 6, 24, 48, 72, and 96 hr after inoculation. No transcriptional expression of *ToxA* was observed for any of the treatments at 0 and 6 hr postinoculation (Figure 6). Expression of *ToxA* in LDN inoculated with Sn2000 peaked at 24 hr postinoculation and from there declined through the 96 hr time point. Expression of *ToxA* in 86-124-treated LDN plants also peaked at 24 hr postinoculation and was detectable at the 48 and 96 hr time points as well, but the relative amounts were less than a tenth of the amount of *ToxA* transcription observed in the Sn2000-inoculated LDN plants (Figure 6).

No expression of *ToxA* in 86–124-inoculated LDN-DIC 5B plants was detected. Small amounts of *ToxA* transcript were detected at the 72 and 96 hr time points in the Sn2000-inoculated LDN-DIC 5B plants, but the amounts were about a hundredth of the amount of *ToxA* transcribed in the Sn2000-inoculated LDN plants (Figure 6).

## Discussion

### Role of Tsc2-Ptr ToxB in tan spot susceptibility in durum wheat

One objective of this research was to determine the role of the *Tsc2*-Ptr ToxB interaction in conferring tan spot susceptibility in a tetraploid durum wheat population. Friesen and Faris (2004) first mapped the Ptr ToxB sensitivity gene *Tsc2* on chromosome arm 2BS using the ITMI population, which was developed from a cross between the synthetic hexaploid wheat W-7984 and the hard red spring wheat variety Opata 85 (PI 591776). W-7984 was synthesized from crossing Altar 84 with the *Aegilops tauschii* accession CI 18 (WPI 219). Because Altar 84 donated the B-genome chromosomes to W-7984, it also donated the *Tsc2* locus, thus rendering W-7984 sensitive to Ptr ToxB. QTL analysis revealed a major QTL on 2BS explaining 69% of the variation and corresponding to the *Tsc2* locus, thus indicating that the *Tsc2*-ToxB interaction was significantly associated with development of the disease in the ITMI population. Other studies have also demonstrated that the *Tsc2* gene is a major susceptibility factor in hexaploid wheat (Abeysekara *et al.* 2010; Singh *et al.* 2010).

Although the durum variety Altar 84 contributed the dominant *Tsc2* allele for Ptr ToxB sensitivity to the synthetic wheat W-7984, the evaluation of the effects of the *Tsc2*-Ptr ToxB interaction were conducted in a hexaploid wheat background (Friesen and Faris 2004). Therefore, in this study, we chose to evaluate a tetraploid population derived from Altar 84 and LDN to determine the effects of the *Tsc2*-Ptr ToxB interaction in a true tetraploid wheat background. The results indicated that the *Tsc2* explained up to 26% of the disease variation. This is the first study to demonstrate that the *Tsc2*-Ptr ToxB interaction plays a significant role in conferring susceptibility in tetraploid wheat, just as it does in hexaploid wheat.

### Role of Tsn1-ToxA in tan spot

Another objective of this study was to evaluate the role of the *Tsn1*-ToxA interaction in conferring tan spot susceptibility in tetraploid wheat. Most of the previous tan spot studies pertaining to the *Tsn1*-ToxA interaction have been conducted in hexaploid wheat (Faris *et al.* 2013 for review), and those studies indicated that the *Tsn1*-ToxA interaction could play a major role (Tomas and Bockus 1987; Lamari and Bernier 1989; Cheong *et al.* 2004; Singh *et al.* 2010), a minor role (Friesen *et al.* 2003; Chu *et al.* 2008a; S. Singh *et al.* 2008; Faris *et al.* 2012), or have no effect (Faris and Friesen 2005), depending on the genetic background. Three studies prior to the current one involved the evaluation of the *Tsn1*-ToxA interaction in conferring tan spot susceptibility in tetraploid wheat. In one study, Chu *et al.* (2010a) evaluated a tetraploid wheat doubled haploid population derived from a cross between the durum variety Lebsock and accession PI 94749 of *T. turgidum* ssp. *carthlicum* (LP population) for reaction to the race 2 isolate 86-124 and the race 1 isolate Pti2, which both produce ToxA. Although the population segregated for reaction to ToxA infiltrations, sensitivity to ToxA had no effect on disease. Further QTL analysis showed no significance for the *Tsn1* locus on 5BL for reaction to either 86–124 or Pti2. In the second tetraploid wheat study, Chu *et al.* (2008c) evaluated 172 accessions of wild emmer wheat (*T. dicoccoides*) for reaction to infiltrations of ToxA and inoculations with the *P. tritici-repentis* race 1 isolate Pti2. They reported a weak (*R*^2^ = 0.03), albeit significant, association between ToxA sensitivity and tan spot susceptibility. Similarly, in the third study, Chu *et al.* (2008b) evaluated 688 accessions of tetraploid wheat subspecies with Pti2 and found that the *Tsn1*-ToxA interaction was not associated with tan spot susceptibility.

The results of the current study agree with those of Chu *et al.* (2008b,c, 2010a), in that the *Tsn1*-ToxA interaction plays little or no role in the development of tan spot in tetraploid wheat. Consistent results were observed when evaluating two different populations (AL and LD5B) and using two different race 2 isolates (86–124 and L13-35) on the AL population. We also conducted single-replication inoculations of the AL population with the race 1 isolates Pti-2 and ASC1, both of which produce ToxA, and found that neither indicated a significant association with the *Tsn1* locus (data not shown). To our knowledge, no study has demonstrated *Tsn1* to play a major role in tan spot susceptibility in tetraploid wheat.

The reasons for varying levels of significance of the *Tsn1*-ToxA interaction in different wheat genetic backgrounds are unknown. It is unlikely that the differences are due to structural variation in the *ToxA* gene among isolates because first, Friesen *et al.* (2006) evaluated ToxA haplotypes of 54 *P. tritici-repentis* isolates, and found no sequence variation in the *ToxA* gene, and second, the same race 2 isolate (86–124) has been used in many of the above mentioned studies and has been associated with variable results including a strong role for the *Tsn1*-ToxA interaction (Lamari and Bernier 1989), a minor role for the interaction (Friesen *et al.* 2003; Chu *et al.* 2010b; Faris *et al.* 2012), and no role for the interaction such as reported in Faris and Friesen (2005), Chu *et al.* (2010a), and the current study.

Faris *et al.* (2011) showed that differential expression of the *ToxA* gene in different isolates of *Pa. nodorum* correlated with the level of SNB susceptibility. This might explain differences in the significance of the *Tsn1*-ToxA interaction among different *P. tritici-repentis* isolates. For example, Faris *et al.* (2012) showed that isolates ASC1, Pti2, and 86-124 explained 5, 22, and 30% of the variation, respectively, even though they all contain the same *ToxA* haplotype (Friesen *et al.* 2006). However, the same explanation for differences in *Tsn1*-ToxA relevance among studies that employed the same isolate, 86-124, would not be valid. In this case, differences in host genetic backgrounds must be responsible. This is in line with the results of a recent study by Manning and Ciuffetti (2015), who reported that the *Tsn1*-ToxA interaction may be epistatic to the production of other NEs, depending on the genetic background of the sensitive host. Perhaps some wheat genotypes possess factors that lead to altered expression levels of the *ToxA* gene through epistasis, or in some way inhibit the recognition of ToxA by *Tsn1* in plants inoculated with fungal spores, but not in plants infiltrated with ToxA. Work is ongoing to elucidate these putative factors and their mechanisms.

### Role of Tsn1-ToxA in SNB

Although this research indicated that the *Tsn1*-ToxA interaction played no significant role in the development of tan spot caused by ToxA-producing isolates of *P. tritici-repentis* in either the AL or the LD5B population, the interaction was highly significant in the development of SNB caused by *Pa. nodorum* in both populations. In previous research, we showed that in the hexaploid wheat BG population the *Tsn1*-ToxA interaction explained as much as 62% of the disease variation for resistance to *Pa. nodorum*, but had no significant association with tan spot caused by ToxA-producing *P. tritici-repentis* isolates (Faris and Friesen 2005; Liu *et al.* 2006). We obtained similar results using the tetraploid LP population where the *Tsn1*-ToxA interaction accounted for 31% of the variation in the development of SNB, but was not associated with tan spot. Therefore, the results of these studies agree with our findings in the AL and LD5B populations. On the contrary, the *Tsn1*-ToxA interaction was evaluated for both tan spot and SNB in one other hexaploid wheat population and found to be significantly associated with both diseases, accounting for 17% of the tan spot variation and 28% of the SNB variation (Chu *et al.* 2008a, 2010b).

Collectively, the currently available data indicate that the role of the *Tsn1*-ToxA interaction in conferring susceptibility to tan spot in hexaploid wheat can range from none to highly important (see Faris *et al.* 2013 for review), and for susceptibility to tan spot in tetraploid wheat the interaction is either not significant or can play a very weak role at most (Chu *et al.* 2008b,c, 2010a; current study). However, research to date indicates that the *Tsn1*-ToxA interaction always plays a highly important role in conferring susceptibility to SNB in both hexaploid and tetraploid wheat when ToxA-producing isolates are used (Abeysekara *et al.* 2012; Chu *et al.* 2008b,c, 2010b; Faris and Friesen 2009; Faris *et al.* 2010, 2011; Friesen *et al.* 2006, 2007, 2008, 2009, 2012; Liu *et al.* 2006).

Differential reactions to ToxA-expressing *P. tritici-repentis* isolates are likely due to different host genetic factors as discussed above, but the reasons for the varying levels of significance of the *Tsn1*-ToxA interaction in causing tan spot compared to SNB are most likely different. Including this research, there have now been four wheat populations (BG, LP, AL, and LD5B) that have shown that the *Tsn1*-ToxA interaction played no role in the development of tan spot, but a major role in the development of SNB. Because each of these studies evaluated disease produced by ToxA-producing isolates of both *P. tritici-repentis* and *Pa. nodorum* on the same population, the differences observed in the effects of the *Tsn1*-ToxA interaction must be due to differences in the biology of the pathogens, *i.e.*, *P. tritici-repentis vs. Pa. nodorum*. In line with this, our expression studies indicated that *Pa. nodorum* expressed *ToxA* at much higher levels than *P. tritici-repentis* on *Tsn1*-containing plants, which is a result reminiscent of those of Faris *et al.* (2011) and Phan *et al.* (2016) who both showed that levels of NE expression in *Pa. nodorum* were strongly correlated with levels of disease.

The reason for the differential expression is not known, but as mentioned above, the *ToxA* gene has existed in *Pa. nodorum* for a very long time and was only recently transferred to *P. tritici-repentis* (Friesen *et al.* 2006). It is possible that *ToxA* functions more efficiently in *Pa. nodorum* compared to *P. tritici-repentis*, perhaps due to the presence of additional and/or different transcription factors that may enhance *ToxA* expression. It is perceivable that transcriptional upregulation of *ToxA* in *Pa. nodorum*-infected plants may occur due to host recognition signals. In other words, pathogen acknowledgment of host recognition, *i.e.*, the presence of *Tsn1*, may lead to upregulation of *ToxA* expression. The fact that *ToxA* expression in the resistant line LDN-DIC 5B was undetectable at 24 hr postinoculation, and levels of *ToxA* expression in the susceptible line LDN were extremely high at the same time point, would suggest that could be the case. Therefore, it seems that *Pa. nodorum* might possess *ToxA*-regulating factors that *P. tritici-repentis* does not. More research is needed to test this hypothesis.

### Conclusions

In conclusion, the *Tsc2*-Ptr ToxB interaction played a significant role in the development of tan spot caused by the Ptr ToxB-producing race 5 isolate DW5. This was the first study to demonstrate this for tetraploid wheat. Therefore, durum wheat breeders should determine whether or not their material possesses the *Tsc2* gene and strive to remove it from their lines using marker-assisted selection. Diagnostic markers for *Tsc2* have been developed and proven to be useful for such purposes (Abeysekara *et al.* 2010). Second, this research showed that the *Tsn1*-ToxA interaction was not associated with the development of tan spot in two tetraploid wheat populations, however, it played a significant role in the development of SNB in both populations. This is the second study to show this result in tetraploid wheat, and validates work in a different tetraploid wheat population (Chu *et al.* 2010a; Friesen *et al.* 2012). Although *Tsn1* may not be relevant for susceptibility to tan spot in these two durum wheat populations, this result needs to be confirmed in other tetraploid wheat populations as well. Regardless of whether or not *Tsn1* is important for tan spot susceptibility in any durum wheat genotype, breeders should still strive to remove *Tsn1* from their materials in an effort to eliminate SNB susceptibility loci and render their lines more resistant. The *Tsn1* gene has been cloned, and numerous diagnostic markers are available for this purpose (Zhang *et al.* 2009; Faris *et al.* 2010). More research is needed to determine why the relevance of the *Tsn1*-ToxA interaction in the development of tan spot is affected by different host genetic backgrounds, and why the interaction is more significant in the development of SNB than tan spot.

## Supplementary Material

Supplemental Material
