# Supplementary material for: New Insights into the Roles of Host Gene-Necrotrophic Effector Interactions in Governing Susceptibility of Durum Wheat to Tan Spot and Septoria nodorum Blotch
Source: G3 (Bethesda). 2016 Oct 24;6(12):4139–50. doi: 10.1534/g3.116.036525 (PMC5144982; doi:10.1534/g3.116.036525)
Supplement: Supplemental Material [file supp_6_12_4139__index.html]

New Insights into the Roles of Host Gene-Necrotrophic Effector Interactions in Governing Susceptibility of Durum Wheat to Tan Spot and Septoria nodorum Blotch — Supplemental Material 

# New Insights into the Roles of Host Gene-Necrotrophic Effector Interactions in Governing Susceptibility of Durum Wheat to Tan Spot and Septoria nodorum Blotch

## Supplemental Material for Faris *et al.*, 2016

**Files in this Data Supplement:**

- Table S1 - Molecular markers, their chromosome assignments, centiMorgan positions, and deviation from expected segregation ratios in the AL population. (.pdf, 295 KB)
