# Supplementary material for: New Insights into the Roles of Host Gene-Necrotrophic Effector Interactions in Governing Susceptibility of Durum Wheat to Tan Spot and Septoria nodorum Blotch
Source: G3 (Bethesda). 2016 Oct 24;6(12):4139–50. doi: 10.1534/g3.116.036525 (PMC5144982; doi:10.1534/g3.116.036525)
Supplement: Supplemental Material [file supp_g3.116.036525_TableS1.pdf]

SUPPLEMENTARY MATERIALS

Table S1. Molecular markers, their chromosome assignments, centiMorgan positions, and deviation from expected segregation ratios in the AL population. Markers in bold are simple sequence repeat (SSR) markers and all others are single nucleotide polymorphism (SNP) markers.

		Marker	Chromosome	Position	Deviation from 1:1 segregation
1	1	***Xcfa2153***	1A	0	ns
2	2	*Xiwa8622*	1A	1.78621	ns
3	3	*Xiwa5150*	1A	2.15385	ns
4	4	*Xiwa6649*	1A	2.52148	ns
5	5	*Xiwa4351*	1A	2.52148	ns
6	6	*Xiwa145*	1A	37.7337	ns
7	7	*Xiwa3435*	1A	37.7337	ns
8	8	*Xiwa8482*	1A	37.7337	ns
9	9	*Xiwa6042*	1A	37.7337	ns
10	10	*Xiwa3805*	1A	37.7337	ns
11	11	*Xiwa3434*	1A	37.7337	ns
12	12	*Xiwa8223*	1A	41.2549	ns
13	13	*Xiwa6934*	1A	41.2549	ns
14	14	*Xiwa4537*	1A	41.2549	ns
15	15	*Xiwa4080*	1A	41.2549	ns
16	16	*Xiwa531*	1A	41.2549	ns
17	17	*Xiwa2056*	1A	66.2672	ns
18	18	*Xiwa6217*	1A	67.3785	ns
19	19	***Xwmc24***	1A	67.3785	ns
20	20	*Xiwa3374*	1A	70.872	ns
21	21	*Xiwa3373*	1A	70.872	ns
22	22	*Xiwa3375*	1A	70.872	ns
23	23	*Xiwa8236*	1A	82.119	ns
24	24	*Xiwa1991*	1A	82.119	ns
25	25	*Xiwa8394*	1A	82.119	ns
26	26	*Xiwa7505*	1A	82.119	ns
27	27	*Xiwa6887*	1A	82.119	ns
28	28	*Xiwa2651*	1A	82.119	ns
29	29	*Xiwa2438*	1A	82.119	ns
30	30	*Xiwa393*	1A	82.119	ns
31	31	*Xiwa392*	1A	82.119	ns
32	32	*Xiwa357*	1A	82.119	ns
33	33	*Xiwa3665*	1A	84.3932	ns

34	34	*Xiwa3806*	1A	84.3932	ns
35	35	*Xiwa3957*	1A	84.3932	ns
36	36	*Xiwa5011*	1A	84.3932	ns
37	37	*Xiwa498*	1A	84.3932	ns
38	38	*Xiwa3160*	1A	84.3932	ns
39	39	***Xbarc28.1***	1A	86.2009	ns
40	40	***Xcfa2135***	1A	88.4747	ns
41	41	*Xiwa7898*	1A	101.008	ns
42	42	*Xiwa2598*	1A	101.008	ns
43	43	*Xiwa2584*	1A	101.008	ns
44	44	*Xiwa1594*	1A	101.008	ns
45	45	*Xiwa6709*	1A	101.372	ns
46	46	*Xiwa8070*	1A	101.372	ns
47	47	*Xiwa5839*	1A	101.372	ns
48	48	*Xiwa6756*	1A	101.372	ns
49	49	*Xiwa4126*	1A	101.372	ns
50	50	*Xiwa4117*	1A	101.372	ns
51	51	*Xiwa4116*	1A	101.372	ns
52	52	*Xiwa268*	1A	101.372	ns
53	53	*Xiwa2152*	1A	101.737	ns
54	54	*Xiwa1615*	1A	101.737	ns
55	55	*Xiwa6835*	1A	101.737	ns
56	56	*Xiwa8523*	1A	128.135	ns
57	57	*Xiwa8528*	1A	128.135	ns
58	58	*Xiwa1710*	1A	128.87	ns
59	59	*Xiwa5407*	1A	128.87	ns
60	60	*Xiwa8020*	1A	130.751	ns
61	61	*Xiwa2035*	1A	132.617	ns
62	62	*Xiwa3089*	1A	132.617	ns
63	63	*Xiwa6458*	1A	132.617	ns
64	64	*Xiwa1559*	1A	132.617	ns
65	65	*Xiwa1560*	1A	132.617	ns
66	66	*Xiwa1558*	1A	132.617	ns
67	67	*Xiwa1557*	1A	132.617	ns
68	68	*Xiwa2317*	1A	134.891	ns
69	69	*Xiwa3409*	1A	144.49	ns
70	1	***Xfcp667(Snn1)***	1B	0	ns
71	2	*Xiwa6787*	1B	28.3216	ns
72	3	*Xiwa4349*	1B	28.3216	ns

73	4	***Xhbe482***	1B	30.2091	ns
74	5	***Xbarc8***	1B	32.6618	ns
75	6	***Xhbg326***	1B	33.1426	*
76	7	*Xiwa7703*	1B	35.8974	ns
77	8	*Xiwa6448*	1B	37.7638	ns
78	9	*Xiwa6450*	1B	37.7638	ns
79	10	*Xiwa8338*	1B	45.2569	ns
80	11	*Xiwa5681*	1B	45.2569	ns
81	12	*Xiwa7219*	1B	45.2569	ns
82	13	*Xiwa5592*	1B	45.2569	ns
83	14	*Xiwa1884*	1B	45.2569	ns
84	15	*Xiwa1451*	1B	45.2569	ns
85	16	*Xiwa580*	1B	45.2569	ns
86	17	*Xiwa284*	1B	45.2569	ns
87	18	***Xbarc137***	1B	47.6624	ns
88	19	***Xgwm18***	1B	47.6624	ns
89	20	***Xhbg419***	1B	48.1128	ns
90	21	***Xgwm11***	1B	48.1128	ns
91	22	***Xcfd59***	1B	48.1128	ns
92	23	***Xgwm264.1***	1B	51.7774	ns
93	24	*Xiwa43*	1B	55.1913	*
94	25	*Xiwa44*	1B	55.1913	*
95	26	*Xiwa107*	1B	55.5536	*
96	27	*Xiwa378*	1B	55.9159	ns
97	28	*Xiwa8081*	1B	61.0927	ns
98	29	*Xiwa7119*	1B	61.0927	ns
99	30	*Xiwa6063*	1B	61.0927	ns
100	31	*Xiwa5348*	1B	61.0927	ns
101	32	*Xiwa7723*	1B	61.455	ns
102	33	*Xiwa7722*	1B	61.455	ns
103	34	*Xiwa7721*	1B	61.455	ns
104	35	*Xiwa5561*	1B	61.455	ns
105	36	*Xiwa3587*	1B	61.455	ns
106	37	*Xiwa3502*	1B	61.455	ns
107	38	*Xiwa2517*	1B	61.455	ns
108	39	*Xiwa1109*	1B	61.455	ns
109	40	***Xhbg258.1***	1B	64.2099	ns
110	41	***Xgwm131***	1B	65.2408	ns
111	42	*Xiwa128*	1B	67.3036	*
112	43	*Xiwa4681*	1B	87.0849	*
113	44	*Xiwa4680*	1B	87.0849	*

114	45	*Xiwa5070*	1B	88.5668	*
115	46	*Xiwa6945*	1B	88.5668	*
116	47	*Xiwa6134*	1B	89.2967	ns
117	48	***Xhbg262.2***	1B	94.4546	ns
118	49	***Xwmc134***	1B	108.886	ns
119	50	*Xiwa5186*	1B	121.077	ns
120	51	*Xiwa3017*	1B	124.134	ns
121	52	*Xiwa6646*	1B	124.496	ns
122	53	*Xiwa4155*	1B	125.226	ns
123	54	*Xiwa4154*	1B	125.588	ns
124	55	***Xbarc81***	1B	126.051	ns
125	56	***Xgwm153***	1B	126.577	ns
126	57	*Xiwa5749*	1B	127.093	ns
127	58	*Xiwa2064*	1B	127.458	ns
128	59	*Xiwa3097*	1B	130.952	ns
129	60	***Xhbg262.1***	1B	145.461	ns
130	61	***Xwmc44***	1B	146.405	ns
131	62	*Xiwa919*	1B	149.808	ns
132	63	*Xiwa8332*	1B	150.17	ns
133	64	*Xiwa7992*	1B	150.17	ns
134	65	*Xiwa3893*	1B	150.532	ns
135	66	*Xiwa3892*	1B	150.532	ns
136	67	*Xiwa3660*	1B	150.532	ns
137	68	*Xiwa848*	1B	150.532	ns
138	69	*Xiwa846*	1B	150.532	ns
139	70	***Xhbe248***	1B	151.476	ns
140	71	***Xhbg236***	1B	151.476	ns
141	72	***Xhbg443***	1B	157.566	*
142	73	*Xiwa725*	1B	159.84	*
143	74	*Xiwa724*	1B	159.84	*
[redacted]					
144	1	***Xhbg224***	2A	0	*
145	2	*Xiwa1512*	2A	30.0256	ns
146	3	*Xiwa6391*	2A	30.0256	ns
147	4	*Xiwa5423*	2A	30.0256	ns
148	5	*Xiwa5341*	2A	30.0256	ns
149	6	*Xiwa5340*	2A	30.0256	ns
150	7	*Xiwa1511*	2A	30.0256	ns
151	8	*Xiwa6922*	2A	30.3879	ns
152	9	*Xiwa4989*	2A	30.3879	ns

153	10	*Xiwa965*	2A	35.1262	ns
154	11	*Xiwa8513*	2A	38.593	ns
155	12	*Xiwa2434*	2A	54.0162	ns
156	13	*Xiwa2433*	2A	54.0162	ns
157	14	*Xiwa2526*	2A	54.7462	ns
158	15	***Xcfa2201***	2A	57.836	ns
159	16	*Xiwa5240*	2A	79.5999	ns
160	17	*Xiwa313*	2A	79.5999	ns
161	18	*Xiwa8491*	2A	79.5999	ns
162	19	*Xiwa7248*	2A	79.5999	ns
163	20	*Xiwa6369*	2A	79.5999	ns
164	21	*Xiwa5586*	2A	79.5999	ns
165	22	*Xiwa3569*	2A	79.5999	ns
166	23	*Xiwa2259*	2A	79.5999	ns
167	24	*Xiwa2245*	2A	79.5999	ns
168	25	*Xiwa1597*	2A	79.5999	ns
169	26	*Xiwa1256*	2A	79.5999	ns
170	27	*Xiwa994*	2A	79.5999	ns
171	28	*Xiwa424*	2A	79.5999	ns
172	29	*Xiwa343*	2A	79.5999	ns
173	30	*Xiwa314*	2A	79.5999	ns
174	31	*Xiwa690*	2A	80.3298	ns
175	32	*Xiwa581*	2A	81.0598	ns
176	33	*Xiwa8328*	2A	81.4221	ns
177	34	*Xiwa8036*	2A	114.505	ns
178	35	*Xiwa2601*	2A	114.505	ns
179	36	*Xiwa4870*	2A	117.159	ns
180	37	*Xiwa3596*	2A	117.159	ns
181	38	*Xiwa1348*	2A	117.159	ns
182	39	*Xiwa8402*	2A	117.159	ns
183	40	*Xiwa6620*	2A	117.159	ns
184	41	*Xiwa1351*	2A	117.159	ns
185	42	*Xiwa1350*	2A	117.159	ns
186	43	*Xiwa1347*	2A	117.159	ns
187	44	*Xiwa6839*	2A	117.159	ns
188	45	*Xiwa6600*	2A	117.159	ns
189	46	*Xiwa3594*	2A	117.159	ns
190	47	*Xiwa1349*	2A	117.159	ns
191	48	*Xiwa319*	2A	117.159	ns
192	49	*Xiwa2778*	2A	125.642	ns
193	50	*Xiwa4491*	2A	125.642	ns

194	51	*Xiwa4493*	2A	125.642	ns
195	52	*Xiwa5879*	2A	125.642	ns
196	53	*Xiwa6963*	2A	125.642	ns
197	54	*Xiwa7327*	2A	125.642	ns
198	55	*Xiwa1088*	2A	125.642	ns
199	56	*Xiwa7335*	2A	125.642	ns
200	57	*Xiwa8325*	2A	125.642	ns
201	58	*Xiwa2370*	2A	149.744	ns
202	59	*Xiwa5959*	2A	156.284	ns
203	60	***Xwmc181***	2A	162.897	*
204	61	***Xhbg327***	2A	178.679	ns
[redacted]					
205	1	***Xwmc382.2***	2B	0	***
206	2	***Xwmc764***	2B	0.40321	**
207	3	*Xiwa6957*	2B	0.40321	***
208	4	***Xwmc661***	2B	4.25669	**
209	5	*Xiwa2304*	2B	8.11017	*
210	6	***Xhbg216***	2B	11.9636	ns
211	7	***Tsc2***	2B	18.5428	ns
212	8	***Xwmc25***	2B	29.841	ns
213	9	***Xwmc154***	2B	35.2007	ns
214	10	*Xiwa6474*	2B	57.3234	ns
215	11	*Xiwa1360*	2B	57.3234	ns
216	12	*Xiwa1359*	2B	57.3234	ns
217	13	***Xwmc597***	2B	78.0516	*
218	14	*Xiwa6069*	2B	93.2027	ns
219	15	*Xiwa5392*	2B	93.2027	ns
220	16	***Xbarc55***	2B	98.98	ns
221	17	*Xiwa8221*	2B	99.38	ns
222	18	*Xiwa4323*	2B	99.38	ns
223	19	*Xiwa7661*	2B	101.637	ns
224	20	*Xiwa6509*	2B	101.637	ns
225	21	*Xiwa3127*	2B	101.637	ns
226	22	*Xiwa3126*	2B	101.637	ns
227	23	*Xiwa1763*	2B	101.637	ns
228	24	*Xiwa897*	2B	101.637	ns
229	25	*Xiwa10*	2B	104.291	ns
230	26	*Xiwa3924*	2B	104.291	ns
231	27	*Xiwa4894*	2B	104.291	ns
232	28	*Xiwa6075*	2B	106.548	ns

233	29	*Xiwa4102*	2B	106.548	ns
234	30	***Xwmc149.2***	2B	115.836	ns
235	31	*Xiwa2130*	2B	151.361	***
236	32	*Xiwa2131*	2B	151.361	***
237	33	*Xiwa2261*	2B	151.361	***
238	34	*Xiwa469*	2B	151.361	***
239	35	*Xiwa5414*	2B	151.361	***
240	36	*Xiwa4866*	2B	179.552	**
241	37	*Xiwa7996*	2B	182.609	**
242	38	*Xiwa2873*	2B	183.712	***
243	39	*Xiwa3176*	2B	183.712	***
244	40	*Xiwa1040*	2B	188.019	****
245	41	*Xiwa7640*	2B	188.019	****
246	42	*Xiwa5081*	2B	196.003	*****
247	43	*Xiwa7112*	2B	196.003	*****
248	44	*Xiwa7113*	2B	196.003	*****
249	45	*Xiwa571*	2B	196.733	*****
250	46	*Xiwa8589*	2B	196.733	*****
[redacted]					
251	1	*Xiwa4257*	3A	0	ns
252	2	*Xiwa5969*	3A	5.01641	ns
253	3	***Xwmc532***	3A	5.01641	ns
254	4	***Xbarc19***	3A	44.5918	ns
255	5	***Xgwm666.1***	3A	44.5918	ns
256	6	***Xhbg345***	3A	59.1157	ns
257	7	*Xiwa8061*	3A	70.5862	ns
258	8	*Xiwa7476*	3A	70.5862	ns
259	9	*Xiwa6783*	3A	70.5862	ns
260	10	*Xiwa6170*	3A	70.5862	ns
261	11	*Xiwa7355*	3A	70.5862	ns
262	12	*Xiwa5632*	3A	70.5862	ns
263	13	*Xiwa4917*	3A	70.5862	ns
264	14	*Xiwa3929*	3A	70.5862	ns
265	15	*Xiwa3836*	3A	70.5862	ns
266	16	*Xiwa3376*	3A	70.5862	ns
267	17	*Xiwa2751*	3A	70.5862	ns
268	18	*Xiwa2618*	3A	70.5862	ns
269	19	*Xiwa1713*	3A	70.5862	ns
270	20	*Xiwa1019*	3A	70.5862	ns
271	21	*Xiwa234*	3A	72.8604	ns

272	22	*Xiwa4075*	3A	72.8604	ns
273	23	*Xiwa8465*	3A	73.2253	ns
274	24	*Xiwa1507*	3A	75.8995	ns
275	25	*Xiwa1604*	3A	75.8995	ns
276	26	*Xiwa1982*	3A	75.8995	ns
277	27	*Xiwa2925*	3A	75.8995	ns
278	28	*Xiwa3772*	3A	75.8995	ns
279	29	*Xiwa7150*	3A	75.8995	ns
280	30	*Xiwa7970*	3A	75.8995	ns
281	31	*Xiwa2332*	3A	75.8995	ns
282	32	*Xiwa3600*	3A	75.8995	ns
283	33	*Xiwa3771*	3A	75.8995	ns
284	34	*Xiwa4001*	3A	75.8995	ns
285	35	*Xiwa6907*	3A	75.8995	ns
286	36	*Xiwa7564*	3A	75.8995	ns
287	37	*Xiwa7817*	3A	75.8995	ns
288	38	*Xiwa7891*	3A	75.8995	ns
289	39	*Xiwa3198*	3A	79.3933	ns
290	40	*Xiwa6750*	3A	79.3933	ns
291	41	*Xiwa7159*	3A	79.3933	ns
292	42	*Xiwa7877*	3A	79.3933	ns
293	43	*Xiwa2348*	3A	99.6186	ns
294	44	*Xiwa5114*	3A	99.6186	ns
295	45	*Xiwa5601*	3A	99.6186	ns
296	46	*Xiwa5602*	3A	99.6186	ns
297	47	*Xiwa5212*	3A	124.996	ns
298	48	*Xiwa855*	3A	124.996	ns
299	49	*Xiwa3111*	3A	124.996	ns
300	50	*Xiwa5980*	3A	124.996	ns
301	51	*Xiwa7602*	3A	124.996	ns
302	52	***Xcfa2037***	3A	157.471	ns
303	53	***Xcfa2076***	3A	159.972	ns
[redacted]					
304	1	*Xiwa4796*	3B	0	ns
305	2	*Xiwa5202*	3B	1.10306	ns
306	3	*Xiwa7230*	3B	2.20612	ns
307	4	*Xiwa4801*	3B	2.20612	ns
308	5	*Xiwa4800*	3B	2.20612	ns
309	6	*Xiwa5426*	3B	2.56843	ns
310	7	*Xiwa5106*	3B	2.56843	ns

311	8	*Xiwa2908*	3B	3.67148	ns
312	9	***Xhbe383***	3B	3.67148	*
313	10	*Xiwa2037*	3B	18.857	*
314	11	*Xiwa8303*	3B	23.1642	*
315	12	*Xiwa8522*	3B	46.3398	ns
316	13	*Xiwa747*	3B	48.2062	ns
317	14	*Xiwa6238*	3B	50.4633	ns
318	15	*Xiwa5710*	3B	82.2308	ns
319	16	*Xiwa8290*	3B	82.2308	ns
320	17	*Xiwa4218*	3B	82.2308	ns
321	18	*Xiwa8583*	3B	82.2308	ns
322	19	*Xiwa7512*	3B	82.2308	ns
323	20	*Xiwa3306*	3B	82.2308	ns
324	21	*Xiwa3305*	3B	82.2308	ns
325	22	*Xiwa3304*	3B	82.2308	ns
326	23	*Xiwa5775*	3B	82.9661	*
327	24	*Xiwa4653*	3B	82.9661	*
328	25	*Xiwa3018*	3B	83.7014	*
329	26	***Xbarc344***	3B	109.942	ns
330	27	*Xiwa6056*	3B	125.655	*
331	28	*Xiwa6057*	3B	125.655	*
332	29	*Xiwa8354*	3B	125.655	*
333	30	***Xbarc84***	3B	140.347	ns
334	31	*Xiwa3159*	3B	159.395	ns
335	32	*Xiwa8479*	3B	162.452	ns
336	33	*Xiwa5892*	3B	167.19	ns
337	34	*Xiwa8203*	3B	167.19	ns
[redacted]					
338	1	***Xbarc138***	4A	0	ns
339	2	***Xcfd71***	4A	0	ns
340	3	***Xwmc491***	4A	2.70512	ns
341	4	*Xiwa603*	4A	22.9228	ns
342	5	*Xiwa4321*	4A	25.1798	ns
343	6	*Xiwa7521*	4A	27.454	ns
344	7	*Xiwa7537*	4A	27.454	ns
345	8	*Xiwa1320*	4A	29.711	ns
346	9	*Xiwa110*	4A	33.5946	ns
347	10	*Xiwa115*	4A	33.5946	ns
348	11	*Xiwa172*	4A	33.5946	ns
349	12	*Xiwa1341*	4A	33.5946	ns

350	13	*Xiwa2781*	4A	33.5946	ns
351	14	*Xiwa3326*	4A	33.5946	ns
352	15	*Xiwa3541*	4A	33.5946	ns
353	16	*Xiwa3542*	4A	33.5946	ns
354	17	*Xiwa3565*	4A	33.5946	ns
355	18	*Xiwa3845*	4A	33.5946	ns
356	19	*Xiwa5652*	4A	33.5946	ns
357	20	*Xiwa5851*	4A	33.5946	ns
358	21	*Xiwa5975*	4A	33.5946	ns
359	22	*Xiwa6597*	4A	33.5946	ns
360	23	*Xiwa7270*	4A	33.5946	ns
361	24	*Xiwa7271*	4A	33.5946	ns
362	25	*Xiwa8414*	4A	33.5946	ns
363	26	*Xiwa1824*	4A	33.9569	ns
364	27	*Xiwa8416*	4A	37.0139	ns
365	28	*Xiwa232*	4A	37.0139	ns
366	29	*Xiwa126*	4A	37.0139	ns
367	30	*Xiwa3792*	4A	40.071	ns
368	31	*Xiwa2000*	4A	40.071	ns
369	32	*Xiwa7859*	4A	40.071	ns
370	33	*Xiwa7657*	4A	40.071	ns
371	34	*Xiwa7617*	4A	40.071	ns
372	35	*Xiwa7522*	4A	40.071	ns
373	36	*Xiwa7134*	4A	40.071	ns
374	37	*Xiwa7133*	4A	40.071	ns
375	38	*Xiwa7092*	4A	40.071	ns
376	39	*Xiwa6944*	4A	40.071	ns
377	40	*Xiwa6540*	4A	40.071	ns
378	41	*Xiwa4772*	4A	40.071	ns
379	42	*Xiwa4771*	4A	40.071	ns
380	43	*Xiwa3582*	4A	40.071	ns
381	44	*Xiwa3581*	4A	40.071	ns
382	45	*Xiwa3361*	4A	40.071	ns
383	46	*Xiwa3344*	4A	40.071	ns
384	47	*Xiwa3088*	4A	40.071	ns
385	48	*Xiwa1919*	4A	40.071	ns
386	49	*Xiwa109*	4A	40.4333	ns
387	50	*Xiwa1720*	4A	67.5376	ns
388	51	***Xwmc468***	4A	78.1068	ns
389	52	*Xiwa6035*	4A	78.1068	ns
390	53	*Xiwa7653*	4A	88.6964	ns

391	54	***Xbarc170***	4A	99.9864	ns
392	55	*Xiwa3068*	4A	128.57	ns
[redacted]					
393	1	*Xiwa506*	4B	0	ns
394	2	*Xiwa2126*	4B	7.07107	ns
395	3	*Xiwa4569*	4B	9.72492	ns
396	4	***Xbarc1045***	4B	44.1718	ns
397	5	***Xgwm113***	4B	45.5234	ns
398	6	*Xiwa7641*	4B	48.6522	**
399	7	*Xiwa58*	4B	48.6522	**
400	8	*Xiwa1007*	4B	63.1988	*
401	9	*Xiwa7752*	4B	64.3184	*
402	10	*Xiwa1035*	4B	64.6833	*
403	11	*Xiwa3396*	4B	65.0483	*
404	12	*Xiwa2031*	4B	90.4256	ns
405	13	*Xiwa2595*	4B	90.4256	ns
406	14	*Xiwa5408*	4B	90.4256	ns
407	15	*Xiwa6465*	4B	90.4256	ns
408	16	*Xiwa7766*	4B	107.219	*
409	17	*Xiwa5520*	4B	107.949	*
[redacted]					
410	1	*Xiwa4767*	5A	0	ns
411	2	*Xiwa4766*	5A	0	ns
412	3	*Xiwa4765*	5A	0	ns
413	4	*Xiwa8155*	5A	13.1371	ns
414	5	*Xiwa8154*	5A	13.1371	ns
415	6	*Xiwa6287*	5A	13.1371	ns
416	7	*Xiwa3190*	5A	13.1371	ns
417	8	*Xiwa7777*	5A	17.082	ns
418	9	*Xiwa1301*	5A	17.082	ns
419	10	*Xiwa2120*	5A	19.3391	ns
420	11	*Xiwa4736*	5A	19.3391	ns
421	12	*Xiwa7351*	5A	19.7014	ns
422	13	*Xiwa5496*	5A	20.0637	ns
423	14	*Xiwa5521*	5A	20.0637	ns
424	15	*Xiwa154*	5A	20.426	ns
425	16	***Xcfa2250***	5A	21.0509	ns
426	17	*Xiwa3445*	5A	21.6759	ns
427	18	*Xiwa333*	5A	21.6759	ns

428	19	*Xiwa2480*	5A	22.4058	ns
429	20	***Xhbg231.2***	5A	28.5585	ns
430	21	***Xbarc360***	5A	31.3778	ns
431	22	*Xiwa2926*	5A	32.5406	ns
432	23	*Xiwa5528*	5A	34.0225	ns
433	24	*Xiwa5735*	5A	36.2795	ns
434	25	*Xiwa7129*	5A	36.6418	ns
435	26	*Xiwa7130*	5A	36.6418	ns
436	27	*Xiwa3975*	5A	38.1236	ns
437	28	*Xiwa5539*	5A	38.1236	ns
438	29	*Xiwa7596*	5A	38.1236	ns
439	30	*Xiwa7597*	5A	38.1236	ns
440	31	*Xiwa7598*	5A	38.1236	ns
441	32	*Xiwa1486*	5A	64.1864	*
442	33	*Xiwa4648*	5A	64.1864	*
443	34	*Xiwa2014*	5A	65.2894	**
444	35	***Xhbg247***	5A	73.424	*****
445	36	*Xiwa2350*	5A	82.8745	*****
446	37	*Xiwa4448*	5A	82.8745	*****
447	38	*Xiwa4449*	5A	82.8745	*****
448	39	*Xiwa7255*	5A	82.8745	*****
449	40	*Xiwa7256*	5A	82.8745	*****
450	41	*Xiwa5040*	5A	83.6044	*****
451	42	*Xiwa509*	5A	85.0863	*****
452	43	*Xiwa3705*	5A	88.9698	*****
453	44	***Xbarc142.2***	5A	90.7722	*****
454	45	*Xiwa3623*	5A	93.0463	*****
455	46	*Xiwa583*	5A	93.4113	*****
456	47	*Xiwa582*	5A	93.4113	*****
457	48	***Xcfa2141***	5A	93.9607	*****
458	49	*Xiwa2113*	5A	113.559	****
459	50	*Xiwa2856*	5A	113.559	****
460	51	*Xiwa2857*	5A	113.559	****
461	52	*Xiwa2858*	5A	113.559	****
462	53	*Xiwa2859*	5A	113.559	****
463	54	*Xiwa4238*	5A	113.559	****
464	55	*Xiwa7009*	5A	113.559	****
465	56	*Xiwa7568*	5A	121.542	*
466	57	*Xiwa1670*	5A	122.272	*
467	58	*Xiwa7880*	5A	130.755	ns

468	1	***Xgwm234***	5B	0	***
469	2	*Xiwa757*	5B	2.83296	*****
470	3	*Xiwa868*	5B	2.83296	*****
471	4	*Xiwa3606*	5B	8.45618	*****
472	5	*Xiwa3607*	5B	8.45618	*****
473	6	*Xiwa332*	5B	13.1944	****
474	7	*Xiwa420*	5B	13.1944	****
475	8	***Xwmc149.1***	5B	17.0478	*
476	9	***Xbarc4***	5B	29.358	***
477	10	***Xgwm544***	5B	29.8387	**
478	11	***Xwmc73***	5B	31.2953	**
479	12	*Xiwa7613*	5B	35.0758	**
480	13	*Xiwa6846*	5B	35.0758	**
481	14	***Xhbe478***	5B	40.4884	***
482	15	***Xbarc216***	5B	40.4884	***
483	16	*Xiwa1342*	5B	41.3975	****
484	17	*Xiwa1965*	5B	41.3975	****
485	18	*Xiwa4377*	5B	41.7599	****
486	19	*Xiwa6816*	5B	41.7599	****
487	20	*Xiwa6521*	5B	41.7599	****
488	21	*Xiwa5494*	5B	41.7599	****
489	22	*Xiwa5079*	5B	41.7599	****
490	23	*Xiwa4378*	5B	41.7599	****
491	24	*Xiwa1176*	5B	41.7599	****
492	25	*Xiwa7857*	5B	42.4898	***
493	26	*Xiwa6568*	5B	42.4898	***
494	27	*Xiwa6567*	5B	42.4898	***
495	28	*Xiwa6447*	5B	42.4898	***
496	29	*Xiwa6429*	5B	42.4898	***
497	30	*Xiwa5334*	5B	42.4898	***
498	31	*Xiwa4708*	5B	42.4898	***
499	32	*Xiwa4686*	5B	42.4898	***
500	33	*Xiwa894*	5B	42.4898	***
501	34	*Xiwa620*	5B	42.4898	***
502	35	*Xiwa3870*	5B	43.5929	***
503	36	***Xhbg279.1***	5B	45.4984	**
504	37	***Xgwm213***	5B	52.2804	***
505	38	***Xbarc74***	5B	53.2905	*
506	39	***Xhbg231.1***	5B	53.2905	*
507	40	*Xiwa7227*	5B	55.196	**

508	41	*Xiwa6689*	5B	55.196	**
509	42	*Xiwa6526*	5B	55.196	**
510	43	*Xiwa5280*	5B	55.196	**
511	44	*Xiwa5279*	5B	55.196	**
512	45	*Xiwa303*	5B	55.196	**
513	46	*Xiwa4422*	5B	55.9259	*
514	47	*Xiwa8603*	5B	57.4078	ns
515	48	*Xiwa5742*	5B	59.6648	*
516	49	***Xgwm371***	5B	69.3578	**
517	50	*Xiwa6638*	5B	74.8245	**
518	51	*Xiwa5486*	5B	74.8245	**
519	52	*Xiwa5331*	5B	74.8245	**
520	53	*Xiwa3436*	5B	74.8245	**
521	54	*Xiwa1380*	5B	74.8245	**
522	55	*Xiwa2536*	5B	75.9357	*
523	56	*Xiwa6867*	5B	79.0163	**
524	57	*Xiwa6468*	5B	79.0163	**
525	58	*Xiwa6383*	5B	79.0163	**
526	59	*Xiwa5283*	5B	79.0163	**
527	60	*Xiwa4074*	5B	79.0163	**
528	61	*Xiwa3985*	5B	79.0163	**
529	62	*Xiwa2455*	5B	79.0163	**
530	63	*Xiwa2454*	5B	79.0163	**
531	64	*Xiwa2453*	5B	79.0163	**
532	65	*Xiwa7795*	5B	79.3786	**
533	66	*Xiwa265*	5B	79.3786	**
534	67	***Xgwm499***	5B	80.3888	**
535	68	*Xiwa7776*	5B	97.4679	ns
536	69	*Xiwa5214*	5B	102.645	ns
537	70	*Xiwa7024*	5B	116.769	ns
538	71	***Tsn1***	5B	117.169	ns
539	72	*Xiwa6915*	5B	117.976	ns
540	73	*Xiwa8375*	5B	119.842	ns
541	74	*Xiwa2306*	5B	119.842	ns
542	75	*Xiwa3002*	5B	120.945	ns
543	76	*Xiwa2255*	5B	122.427	ns
544	77	*Xiwa2565*	5B	122.427	ns
545	78	*Xiwa6024*	5B	122.427	ns
546	79	*Xiwa6671*	5B	122.427	ns
547	80	*Xiwa1775*	5B	122.427	ns
548	81	*Xiwa3226*	5B	122.789	ns

549	82	***Xgwm408***	5B	127.004	ns
550	83	*Xiwa2500*	5B	141.87	ns
551	84	***Xgwm604***	5B	143.625	ns
552	85	***Xhbg399***	5B	145.92	ns
553	86	***Xbarc142.1***	5B	147.284	ns
554	87	*Xiwa7872*	5B	148.6	ns
555	88	*Xiwa7478*	5B	148.6	ns
556	89	*Xiwa4539*	5B	148.6	ns
557	90	*Xiwa6779*	5B	152.067	ns
558	91	*Xiwa8250*	5B	152.067	ns
559	92	***Xwmc160***	5B	153.837	ns
560	93	***Xbarc232***	5B	154.746	ns
561	94	***Xwmc235.1***	5B	158.287	ns
562	95	*Xiwa7966*	5B	160.647	ns
563	96	*Xiwa2388*	5B	160.647	ns
564	97	*Xiwa7965*	5B	160.647	ns
565	98	***Xhbg429***	5B	179.084	**
566	99	*Xiwa3972*	5B	180.887	*
567	100	*Xiwa6393*	5B	180.887	*
568	101	***Xwmc640***	5B	185.354	**
569	102	***Xbarc243***	5B	203.623	**
570	103	*Xiwa3658*	5B	210.598	ns
571	104	*Xiwa2093*	5B	211.717	ns
572	1	***Xhbe297***	6A	0	ns
573	2	***Xmag1885***	6A	34.1604	**
574	3	*Xiwa7572*	6A	73.9446	ns
575	4	*Xiwa6853*	6A	73.9446	ns
576	5	*Xiwa6116*	6A	73.9446	ns
577	6	*Xiwa4603*	6A	73.9446	ns
578	7	*Xiwa4602*	6A	73.9446	ns
579	8	*Xiwa504*	6A	73.9446	ns
580	9	*Xiwa3488*	6A	76.2016	ns
581	10	*Xiwa8568*	6A	76.2016	ns
582	11	*Xiwa6858*	6A	76.2016	ns
583	12	*Xiwa4478*	6A	76.2016	ns
584	13	*Xiwa3487*	6A	76.2016	ns
585	14	*Xiwa2481*	6A	77.6835	ns
586	15	*Xiwa8602*	6A	87.1977	**
587	16	*Xiwa20*	6A	93.7383	ns

588	17	*Xiwa19*	6A	93.7383	ns
589	18	***Xhbg416***	6A	116.334	ns
590	19	*Xiwa399*	6A	118.945	ns
591	20	*Xiwa8592*	6A	119.307	ns
592	21	*Xiwa6288*	6A	120.41	ns
593	22	*Xiwa6247*	6A	120.41	ns
594	23	*Xiwa6033*	6A	120.41	ns
595	24	*Xiwa6012*	6A	120.41	ns
596	25	*Xiwa664*	6A	120.772	ns
597	26	*Xiwa4865*	6A	121.876	ns
598	27	*Xiwa6596*	6A	121.876	ns
599	28	*Xiwa6699*	6A	121.876	ns
600	29	*Xiwa2895*	6A	121.876	ns
601	30	*Xiwa4036*	6A	121.876	ns
602	31	*Xiwa4035*	6A	121.876	ns
603	32	*Xiwa2192*	6A	121.876	ns
604	33	*Xiwa1514*	6A	121.876	ns
605	34	*Xiwa1475*	6A	121.876	ns
606	35	*Xiwa1474*	6A	121.876	ns
607	36	*Xiwa1194*	6A	121.876	ns
608	37	*Xiwa741*	6A	121.876	ns
609	38	*Xiwa651*	6A	121.876	ns
610	39	*Xiwa650*	6A	121.876	ns
611	40	*Xiwa218*	6A	121.876	ns
612	41	*Xiwa4928*	6A	121.876	ns
613	42	*Xiwa1671*	6A	121.876	ns
614	43	*Xiwa1235*	6A	121.876	ns
615	44	*Xiwa4929*	6A	121.876	ns
616	45	*Xiwa8585*	6A	122.611	ns
617	46	*Xiwa7052*	6A	123.341	ns
[redacted]					
618	1	*Xiwa1254*	6B	0	ns
619	2	*Xiwa8314*	6B	0.72994	ns
620	3	*Xiwa4633*	6B	4.61346	ns
621	4	*Xiwa7725*	6B	20.0366	**
622	5	*Xiwa52*	6B	23.5035	*
623	6	***Xcfd13***	6B	24.5788	***
624	7	*Xiwa3229*	6B	26.7778	**
625	8	*Xiwa7239*	6B	45.7779	ns
626	9	*Xiwa861*	6B	45.7779	ns

627	10	*Xiwa8134*	6B	48.0349	*
628	11	*Xiwa5888*	6B	48.0349	*
629	12	*Xiwa3411*	6B	48.0349	*
630	13	*Xiwa3410*	6B	48.0349	*
631	14	*Xiwa7808*	6B	49.5168	*
632	15	*Xiwa7810*	6B	49.5168	*
633	16	*Xiwa7809*	6B	49.5168	*
634	17	*Xiwa7807*	6B	49.5168	*
635	18	*Xiwa7618*	6B	49.5168	*
636	19	*Xiwa2219*	6B	49.5168	*
637	20	*Xiwa5056*	6B	50.2467	*
638	21	*Xiwa5055*	6B	50.2467	*
639	22	*Xiwa7937*	6B	53.7136	*
640	23	*Xiwa7689*	6B	53.7136	*
641	24	*Xiwa3501*	6B	53.7136	*
642	25	*Xiwa7897*	6B	54.4435	ns
643	26	*Xiwa7896*	6B	54.4435	ns
644	27	*Xiwa2307*	6B	54.4435	ns
645	28	*Xiwa8011*	6B	59.6621	**
646	29	*Xiwa4078*	6B	59.6621	**
647	30	*Xiwa7676*	6B	60.7651	**
648	31	*Xiwa7783*	6B	60.7651	**
649	32	*Xiwa7401*	6B	60.7651	**
650	33	*Xiwa6293*	6B	60.7651	**
651	34	*Xiwa5748*	6B	60.7651	**
652	35	*Xiwa4515*	6B	60.7651	**
653	36	*Xiwa4065*	6B	60.7651	**
654	37	*Xiwa3971*	6B	60.7651	**
655	38	*Xiwa2451*	6B	60.7651	**
656	39	*Xiwa1742*	6B	60.7651	**
657	40	*Xiwa1545*	6B	60.7651	**
658	41	*Xiwa685*	6B	60.7651	**
659	42	*Xiwa4986*	6B	60.7651	**
660	43	*Xiwa6153*	6B	61.8682	*
661	44	*Xiwa5504*	6B	61.8682	*
662	45	*Xiwa5102*	6B	61.8682	*
663	46	*Xiwa5029*	6B	61.8682	*
664	47	*Xiwa3917*	6B	61.8682	*
665	48	*Xiwa2780*	6B	61.8682	*
666	49	*Xiwa1839*	6B	61.8682	*
667	50	*Xiwa1838*	6B	61.8682	*

668	51	*Xiwa3652*	6B	62.2331	*
669	52	*Xiwa3797*	6B	62.2331	*
670	53	*Xiwa4169*	6B	62.2331	*
671	54	*Xiwa4848*	6B	62.2331	*
672	55	*Xiwa4924*	6B	62.2331	*
673	56	*Xiwa5966*	6B	62.2331	*
674	57	*Xiwa6101*	6B	62.2331	*
675	58	*Xiwa4170*	6B	62.2331	*
676	59	*Xiwa3459*	6B	62.9631	ns
677	60	*Xiwa8165*	6B	62.9631	ns
678	61	*Xiwa5345*	6B	62.9631	ns
679	62	***Xhbg291***	6B	63.3941	ns
680	63	***Xgwm88***	6B	64.0038	*
681	64	***Xgwm193***	6B	64.0038	*
682	65	*Xiwa4484*	6B	72.5862	ns
683	66	*Xiwa4485*	6B	72.5862	ns
684	67	*Xiwa5722*	6B	73.6974	ns
685	68	*Xiwa6329*	6B	74.0597	ns
686	69	*Xiwa6660*	6B	74.0597	ns
687	70	***Xbarc354***	6B	76.5617	ns
688	71	***Xbarc79***	6B	81.3376	ns
689	72	*Xiwa457*	6B	82.2148	ns
690	73	*Xiwa3967*	6B	83.3179	ns
691	74	*Xiwa6599*	6B	83.3179	ns
692	75	*Xiwa5170*	6B	83.3179	ns
693	76	*Xiwa1473*	6B	83.3179	ns
694	77	*Xiwa1472*	6B	83.3179	ns
695	78	*Xiwa4202*	6B	83.6802	ns
696	79	*Xiwa3769*	6B	83.6802	ns
697	80	*Xiwa1531*	6B	83.6802	ns
698	81	*Xiwa225*	6B	84.4101	ns
699	82	*Xiwa2346*	6B	84.4101	ns
700	83	*Xiwa8566*	6B	84.4101	ns
701	84	*Xiwa2347*	6B	84.4101	ns
702	85	*Xiwa3636*	6B	84.7724	ns
703	86	*Xiwa221*	6B	85.1374	ns
704	87	*Xiwa4337*	6B	85.1374	ns
705	88	*Xiwa4959*	6B	85.1374	ns
706	89	*Xiwa5148*	6B	85.1374	ns
707	90	*Xiwa967*	6B	104.137	ns
708	91	*Xiwa1629*	6B	108.876	ns

709	92	*Xiwa1628*	6B	108.876	ns
710	93	*Xiwa405*	6B	108.876	ns
711	94	*Xiwa404*	6B	108.876	ns
712	95	***Xgwm219***	6B	111.099	ns
[redacted]					
713	1	*Xiwa7500*	7A	0	ns
714	2	*Xiwa6331*	7A	8.9934	ns
715	3	*Xiwa679*	7A	8.9934	ns
716	4	*Xiwa8390*	7A	32.169	ns
717	5	*Xiwa954*	7A	41.1624	ns
718	6	***Xcfa2049***	7A	42.339	ns
719	7	*Xiwa3505*	7A	47.4188	ns
720	8	***Xhbg469***	7A	53.8256	ns
721	9	***Xhbg238***	7A	80.83	ns
722	10	*Xiwa7472*	7A	109.985	ns
723	11	*Xiwa7724*	7A	109.985	ns
724	12	*Xiwa7792*	7A	109.985	ns
725	13	*Xiwa7990*	7A	109.985	ns
726	14	*Xiwa8171*	7A	109.985	ns
727	15	*Xiwa1477*	7A	110.347	ns
728	16	*Xiwa8073*	7A	122.642	ns
729	17	*Xiwa7090*	7A	124.508	ns
730	18	*Xiwa448*	7A	124.871	ns
731	19	*Xiwa4996*	7A	124.871	ns
732	20	*Xiwa689*	7A	125.233	ns
733	21	*Xiwa808*	7A	125.233	ns
734	22	*Xiwa1502*	7A	125.233	ns
735	23	*Xiwa2385*	7A	125.233	ns
736	24	*Xiwa2386*	7A	125.233	ns
737	25	*Xiwa2387*	7A	125.233	ns
738	26	*Xiwa2437*	7A	125.233	ns
739	27	*Xiwa4062*	7A	125.233	ns
740	28	*Xiwa1277*	7A	142.026	ns
741	29	*Xiwa3863*	7A	142.026	ns
742	30	*Xiwa6124*	7A	142.026	ns
743	31	*Xiwa7554*	7A	142.026	ns
744	32	*Xiwa8066*	7A	142.026	ns
745	33	*Xiwa788*	7A	143.129	ns
746	34	*Xiwa1418*	7A	143.129	ns
747	35	*Xiwa1871*	7A	143.129	ns

748	36	*Xiwa2381*	7A	143.129	ns
749	37	*Xiwa2954*	7A	143.129	ns
750	38	*Xiwa3693*	7A	143.129	ns
751	39	*Xiwa3694*	7A	143.129	ns
752	40	*Xiwa4818*	7A	143.129	ns
753	41	*Xiwa6183*	7A	143.129	ns
754	42	*Xiwa4411*	7A	159.228	ns
755	43	*Xiwa4672*	7A	159.228	ns
756	44	*Xiwa4735*	7A	159.228	ns
757	45	*Xiwa4817*	7A	159.228	ns
758	46	*Xiwa5526*	7A	159.228	ns
759	47	*Xiwa7650*	7A	159.228	ns
760	48	*Xiwa7651*	7A	159.228	ns
761	49	*Xiwa7770*	7A	159.228	ns
762	50	*Xiwa7917*	7A	159.228	ns
763	51	*Xiwa8115*	7A	159.228	ns
764	52	*Xiwa8248*	7A	159.228	ns
765	53	*Xiwa2176*	7A	159.228	ns
766	54	*Xiwa6940*	7A	159.59	ns
767	55	*Xiwa2011*	7A	165.214	ns
768	56	*Xiwa2012*	7A	165.214	ns
769	57	*Xiwa4288*	7A	165.214	ns
770	58	*Xiwa614*	7A	180.792	ns
771	59	*Xiwa2775*	7A	180.792	ns
772	60	*Xiwa2776*	7A	180.792	ns
773	61	*Xiwa5489*	7A	180.792	ns
774	62	***Xcfa2123***	7A	180.792	ns
775	63	*Xiwa1424*	7A	184.968	ns
776	64	*Xiwa1425*	7A	184.968	ns
777	65	*Xiwa1724*	7A	184.968	ns
778	66	*Xiwa1726*	7A	184.968	ns
779	67	*Xiwa5790*	7A	184.968	ns
780	68	*Xiwa6715*	7A	184.968	ns
781	69	*Xiwa7046*	7A	184.968	ns
782	70	***Xcfd20.1***	7A	223.85	ns
783	71	***Xhbg234***	7A	256.007	ns
784	72	*Xiwa7409*	7A	262.405	ns
[redacted]					
785	1	*Xiwa1526*	7B	0	**
786	2	*Xiwa783*	7B	0	**

787	3	*Xiwa1181*	7B	0	**
788	4	***Xgwm537***	7B	16.704	*
789	5	*Xiwa7233*	7B	27.7015	ns
790	6	*Xiwa3572*	7B	27.7015	ns
791	7	*Xiwa3508*	7B	27.7015	ns
792	8	*Xiwa3507*	7B	27.7015	ns
793	9	*Xiwa518*	7B	27.7015	ns
794	10	*Xiwa5566*	7B	29.582	ns
795	11	*Xiwa5565*	7B	29.582	ns
796	12	*Xiwa3958*	7B	29.582	ns
797	13	*Xiwa2832*	7B	29.582	ns
798	14	*Xiwa3959*	7B	29.582	ns
799	15	*Xiwa4873*	7B	46.3752	ns
800	16	*Xiwa3121*	7B	49.8421	ns
801	17	*Xiwa5210*	7B	49.8421	ns
802	18	*Xiwa5661*	7B	49.8421	ns
803	19	*Xiwa7846*	7B	49.8421	ns
804	20	*Xiwa7831*	7B	49.8421	ns
805	21	*Xiwa7830*	7B	49.8421	ns
806	22	*Xiwa6788*	7B	49.8421	ns
807	23	*Xiwa5663*	7B	49.8421	ns
808	24	*Xiwa5662*	7B	49.8421	ns
809	25	*Xiwa3663*	7B	49.8421	ns
810	26	*Xiwa8233*	7B	51.3239	ns
811	27	*Xiwa6401*	7B	62.4689	ns
812	28	*Xiwa3987*	7B	62.4689	ns
813	29	*Xiwa1361*	7B	62.4689	ns
814	30	*Xiwa354*	7B	63.5802	ns
815	31	*Xiwa355*	7B	63.5802	ns
816	32	*Xiwa8232*	7B	68.757	ns
817	33	*Xiwa6322*	7B	68.757	ns
818	34	*Xiwa6667*	7B	68.757	ns
819	35	*Xiwa320*	7B	91.9326	ns
820	36	*Xiwa394*	7B	91.9326	ns
821	37	*Xiwa395*	7B	91.9326	ns
822	38	*Xiwa521*	7B	93.0357	ns
823	39	*Xiwa8519*	7B	93.0357	ns
824	40	*Xiwa520*	7B	93.0357	ns
825	41	*Xiwa2369*	7B	107.801	ns
826	42	*Xiwa6532*	7B	107.801	ns
827	43	*Xiwa130*	7B	110.455	ns

828	44	*Xiwa2191*	7B	112.712	ns
829	45	*Xiwa4864*	7B	112.712	ns
830	46	*Xiwa2193*	7B	113.442	ns
831	47	*Xiwa4749*	7B	113.442	ns
832	48	*Xiwa6246*	7B	113.442	ns
833	49	*Xiwa7907*	7B	114.172	ns
834	50	*Xiwa2389*	7B	115.275	ns
835	51	*Xiwa7260*	7B	116.005	ns
836	52	*Xiwa7964*	7B	116.005	ns
837	53	*Xiwa1091*	7B	116.005	ns
838	54	***Xhbg396***	7B	119.458	ns
839	55	*Xiwa1044*	7B	120.278	ns
840	56	*Xiwa4522*	7B	120.278	ns
841	57	*Xiwa4888*	7B	120.278	ns
842	58	*Xiwa7190*	7B	120.278	ns
843	59	*Xiwa181*	7B	122.144	ns
844	60	*Xiwa1647*	7B	124.401	ns

ns = nonsignificant
* = $P < 0.05$
** = $P < 0.01$
*** = $P < 0.005$
**** = $P < 0.001$
***** = $P < 0.0005$
